# Spine-like Nanostructured Carbon Interconnected by Graphene for High-performance Supercapacitors

**DOI:** 10.1038/srep06118

**Published:** 2014-08-19

**Authors:** Sang-Hoon Park, Seung-Beom Yoon, Hyun-Kyung Kim, Joong Tark Han, Hae-Woong Park, Joah Han, Seok-Min Yun, Han Gi Jeong, Kwang Chul Roh, Kwang-Bum Kim

**Affiliations:** 1 Department of Materials Science and Engineering, Yonsei University, 134 Shinchon-dong, Seodaemoon-gu, Seoul 120-749, Republic of Korea; 2 Nano Carbon Materials Research Group, Korea Electrotechnology Research Institute, Changwon 642-120, Republic of Korea; 3 Energy Efficient Materials Team, Energy & Environmental Division, Korea Institute of Ceramic Engineering & Technology, 233-5 Gasan-dong, Guemcheon-gu, Seoul 153-801, Republic of Korea; 4 VINA Tech, 587-40, Gwahaksaneop 2-ro, Ochang-eup, Cheongwon-gun, Chungcheongbuk-do, 63-883, Republic of Korea

## Abstract

Recent studies on supercapacitors have focused on the development of hierarchical nanostructured carbons by combining two-dimensional graphene and other conductive sp^2^ carbons, which differ in dimensionality, to improve their electrochemical performance. Herein, we report a strategy for synthesizing a hierarchical graphene-based carbon material, which we shall refer to as spine-like nanostructured carbon, from a one-dimensional graphitic carbon nanofiber by controlling the local graphene/graphitic structure *via* an expanding process and a co-solvent exfoliation method. Spine-like nanostructured carbon has a unique hierarchical structure of partially exfoliated graphitic blocks interconnected by thin graphene sheets in the same manner as in the case of ligaments. Owing to the exposed graphene layers and interconnected sp^2^ carbon structure, this hierarchical nanostructured carbon possesses a large, electrochemically accessible surface area with high electrical conductivity and exhibits high electrochemical performance.

Electric double-layer capacitors (EDLCs), also called supercapacitors or ultracapacitors, have attracted considerable interest during the past few decades owing to their high power density, long cycle life and fast recharge capability^1^. Their high power density is attributed to a charge storage mechanism that involves a fast and reversible ion adsorption/desorption reaction at the electrochemical interface between the electrolyte and electrode^2^. To achieve fast and reversible electrochemical interactions, such a charge storage mechanism requires that the electrode material has not only a sufficiently large surface area but also high electrical conductivity^1^,^2^,^3^,^4^.

Among the various electrode materials available for supercapacitors, graphene has attracted considerable interest as a next-generation carbon material owing to its high surface area (~2630 m^2^ g^−1^), superior conductivity and excellent electrochemical stability^5^,^6^,^7^,^8^,^9^,^10^,^11^,^12^,^13^. However, optimizing the electrochemical performance of graphene-based electrode materials remains challenging because the electrochemically active surface area is smaller than that predicted from the ultrahigh surface area of ideal graphene^7^,^8^,^9^,^10^,^11^,^12^,^13^. This is mainly attributed to the facile aggregation/restacking of graphene sheets caused by the strong van der Waals interaction during the electrode fabrication process^9^. Furthermore, the low electrical conductivity of graphene-based electrodes relative to that of ideal graphene also limits the electrochemical performance of graphene-based electrodes.

To solve these problems, recent studies have focused on the synthesis of hierarchical nanostructured carbons by combining two-dimensional graphene and other conductive sp^2^ carbons, which differ in dimensionality, such as carbon black, fullerene, carbon nanotubes (CNTs) and mesoporous carbon^14^,^15^,^16^,^17^,^18^,^19^,^20^,^21^,^22^,^23^,^24^,^25^,^26^. Owing to their multidimensional structure and three-dimensional conductive networks, hierarchical nanostructured carbons possess large, electrochemically active, surface areas with efficient porous channels and high electrical conductivity, which facilitate fast ion diffusion/electron transfer and can enhance the electrochemical performance of a nanostructured electrode^14^,^15^,^16^,^17^,^18^.

To date, the strategies for synthesizing these hierarchical nanostructured carbons have generally concentrated upon either assembling graphene with other pre-synthesized carbon materials or a bottom-up approach involving the growth of carbon materials on the surface of graphene. In the assembly approach, pre-synthesized carbon materials are hierarchically embedded in or wrapped with graphene by controlling the chemical interaction between the carbon materials^17^,^18^,^19^,^20^,^21^,^22^. On the other hand, in the bottom-up approach, carbon materials such as CNTs or mesoporous carbon are grown on the surface of graphene by using chemical vapor deposition (CVD) or soft/hard templates^23^,^24^,^25^,^26^. However, these strategies require additional processes for combining graphene with other carbon materials, and in some cases, contact resistance may occur at the interface between the different types of carbon material.

Herein, we report a strategy for the synthesis of a hierarchical graphene-based carbon material, henceforth referred to as spine-like nanostructured carbon, from one-dimensional graphitic carbon nanofibers (CNFs) by controlling the local graphene/graphitic structure *via* a combination of an expanding process and a co-solvent exfoliation method. The resultant spine-like nanostructured carbon has a unique hierarchical structure of partially exfoliated sp^2^ graphitic blocks interconnected by ligament-like thin graphene nanoplatelets. Using the aforementioned co-solvent method induces only mild exfoliation conditions that prevent these interconnections from breaking. By virtue of the exposed graphene layers and interconnected sp^2^ carbon structure, this hierarchical nanostructured carbon not only possesses a high surface area but also exhibits high electrical conductivity. Consequently, this hierarchical nanostructured carbon exhibits improved electrochemical performance in terms of specific capacitance and high rate capability.

## Results

**Figure 1:**
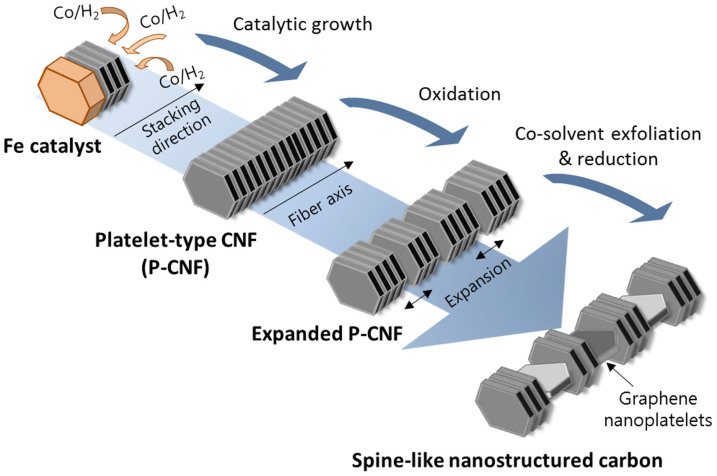
Synthetic route of spine-like nanostructured carbon.

Figure 1 illustrates the proposed synthetic route of the spine-like nanostructured carbon. We initially prepared the platelet-type CNF (P-CNF) by using chemical vapor deposition (CVD) with the assistance of an Fe catalyst^27^,^28^. The as-prepared P-CNF with graphene layers stacked perpendicularly along the fiber axis was then expanded by adjusting the oxidation treatment. As a final step, the expanded P-CNF was partially exfoliated *via* the co-solvent exfoliation method and then chemically reduced to form the spine-like nanostructured carbon.

**Figure 2:**
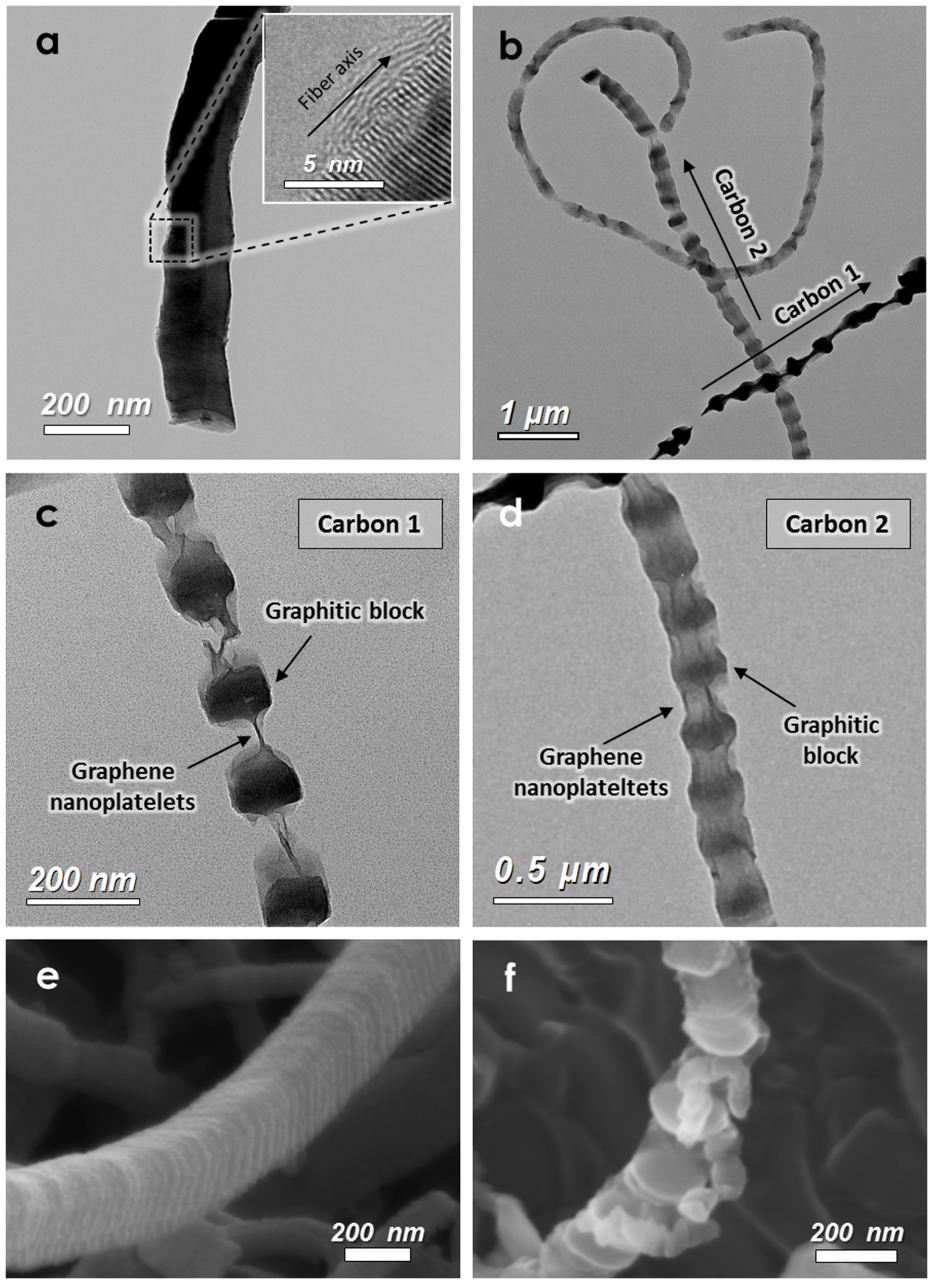
TEM images of (a) as-prepared P-CNF (inset shows a high-magnification view of the edge structure) and (b) spine-like nanostructured carbon synthesized according to Figure 1. (c) High-magnification image of the structure labeled Carbon 1 in (b); (d) high-magnification image of the structure labeled Carbon 2 in (b). SEM images of (e) as-prepared P-CNF and nanostructured carbon.

Figure 2 shows the change in morphology from as-prepared P-CNF to spine-like nanostructured carbon during the synthetic route in Figure 1. The transmission electron microscopy (TEM) image of Figure 2a shows the as-prepared P-CNF with a fiber diameter of ~200 nm. In the high-magnification TEM image (inset in Figure 2a), graphene layers are stacked perpendicular to the fiber axis with a distance of ~0.34 nm and their edges are exposed in the radially outward direction of the fiber. Owing to this stacking direction of the graphene layers, the as-prepared P-CNF could be partially exfoliated along the fiber axis. Figure 2b shows the spine-like nanostructured carbon synthesized from as-prepared P-CNF by the expanding process and the co-solvent exfoliation method. The TEM image reveals that spine-like nanostructured carbon is partially exfoliated but maintains a one-dimensional shape on a microscopic scale. Individual spine-like nanostructured carbons can be distinguished owing to their different orientations on the TEM grid. Figures 1c and 1d present high-magnification TEM images of differently oriented Carbons 1 and 2, respectively. The graphitic blocks of a similar size (~100 nm) in Carbon 1 are individually exfoliated but interconnected by thin graphene nanoplatelets. On the other hand, in Carbon 2, the basal planes of the graphene nanoplatelets are exposed between graphitic blocks (see Supplementary Figure S1 for graphene nanoplatelets at high magnification). Partial exfoliation of the graphitic blocks is also seen by comparing scanning electron microscopy (SEM) images of as-prepared P-CNF (Figure 2e) and spine-like nanostructured carbon (Figure 2f). To our best knowledge, this spine-like nanostructured carbon, composed of a periodically alternating structure of exfoliated graphitic blocks and graphene nanoplatelets, is the first such carbon nanostructure reported.

The TEM and SEM results reveal two important structural features of spine-like nanostructured carbon. First, graphitic blocks of similar size (~100 nm) are periodically exfoliated along the major axis. Second, the exfoliated graphitic blocks are interconnected by thin graphene nanoplatelets. The formation of this hierarchical structure could be governed by the expanding and exfoliating conditions.

The structural periodicity (the graphitic blocks with a similar size) in the nanostructured carbon could be induced by adjusting oxidation condition during expanding process. The average number of stacked graphene nanoplatelets for graphene-based carbon materials such as reduced graphene oxide (RGO, multi-layer graphene) has been reported to be highly affected by the degree of oxidation in the starting material (graphite oxide, GO)^29^. Yoon *et al*. also demonstrated that the size of the exfoliated graphitic blocks could be controlled by the degree of CNF oxidation^28^. In their study, as the degree of CNF oxidation increased, the same exfoliation procedure resulted in thinner exfoliated graphitic blocks. Consistent with this view, CNFs could be periodically expanded with graphitic structures of a similar size by adjusting the oxidation treatment (see Supplementary Figure S2 for the optimized expanding condition).

The conditions during exfoliation may determine the connectivity (interconnected or disconnected) of the graphene nanoplatelets between the graphitic blocks. As noted above, we introduced a co-solvent method to avoid complete exfoliation of the graphene layers and disconnection of the graphitic blocks in the resulting nanostructured carbon. Generally, neutral or basic water and a number of organic solvents (dimethylformamide, N-methyl pyrrolidone and tetrahydrofuran) are used to effectively exfoliate GO (or to de-bundle CNTs) and generate stable dispersions by a sonication or homogenization treatment^30^,^31^,^32^,^33^. In contrast, these functionalized carbons are hardly exfoliated in methanol, ethanol and acetone during sonication owing to their relatively low solubility in these media^31^,^32^,^33^. Here, a mixed solvent of water (soluble) and ethanol (insoluble) lowered the dispersion stability of expanded CNF and yielded partially exfoliated graphene layers that interconnected the graphitic blocks. Although exfoliated graphitic carbon materials have been reported in several studies, the investigations have generally focused on uniform exfoliation of the graphitic structure with little attention to controlling the local graphene/graphitic structure^34^,^35^,^36^. In particular, no attempts have been made yet to control the local nanostructure during exfoliation using a mixture of soluble and insoluble solvents for a functionalized carbon dispersion.

**Figure 3:**
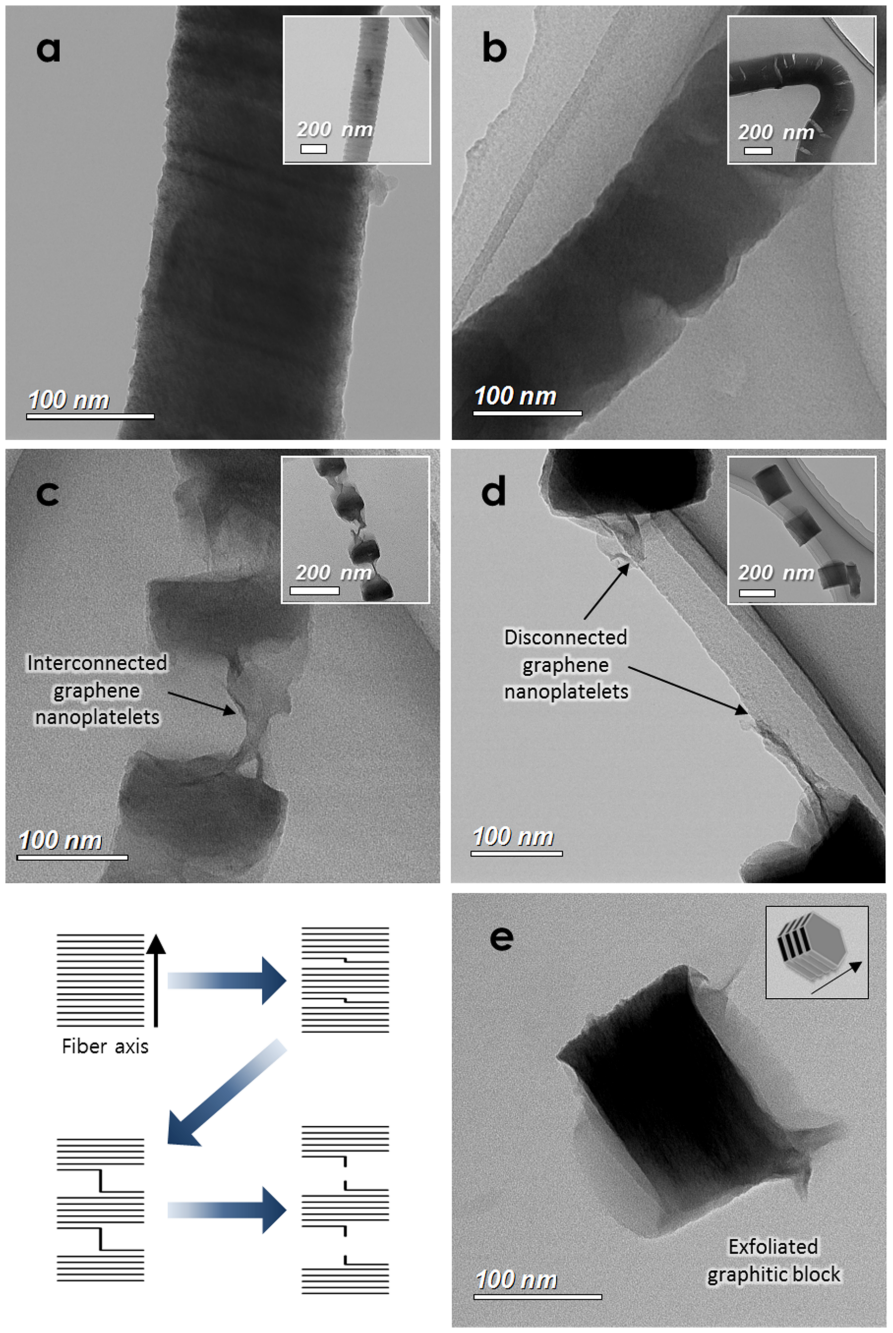
TEM images of exfoliation sequence of graphitic blocks in spine-like nanostructured carbon. (a) As-prepared P-CNF, (b) expanded P-CNF, (c) partially exfoliated graphitic blocks with interconnected graphene layers in spine-like nanostructured carbon, (d) graphitic blocks perfectly separated by a harsh exfoliation process and (e) perfectly separated single graphitic block.

Figure 3 shows the exfoliation sequence of the graphitic blocks in CNFs during synthesis. Compared with the as-prepared P-CNF (Figure 3a), the expanded P-CNF prepared by oxidation treatment (Figure 3b) shows a graphitic structure that is partially widened along the direction of the fiber axis. This effect may be attributed to the weak van der Waals force between the graphene layers due to the oxygen-containing functional groups formed during oxidation treatment^9^,^28^,^29^. Figure 3c shows partially exfoliated graphitic blocks in the spine-like nanostructured carbon prepared from expanded P-CNF by using the co-solvent exfoliation method; the exfoliated graphitic blocks are interconnected by graphene nanoplatelets (shown in the schematic on the bottom left). To investigate the degree of exfoliation and connectivity of the graphene nanoplatelets between the graphitic blocks in the nanostructured carbon, relatively harsh exfoliation conditions were introduced by using water as the soluble solvent for expanded P-CNF. Figure 3d shows that the graphitic blocks in the CNF are fully exfoliated by sonication in water; disconnected graphene layers are seen between the separated graphitic blocks. This result suggests that the co-solvent exfoliation method is useful for synthesizing spine-like nanostructured carbon. A graphitic block fully separated by harsh exfoliation treatment is shown in Figure 3e. The length of a separated single graphitic block (along the initial fiber axis) is ~100 nm, corresponding to the average block size in spine-like nanostructured carbon in Figure 2.

**Figure 4:**
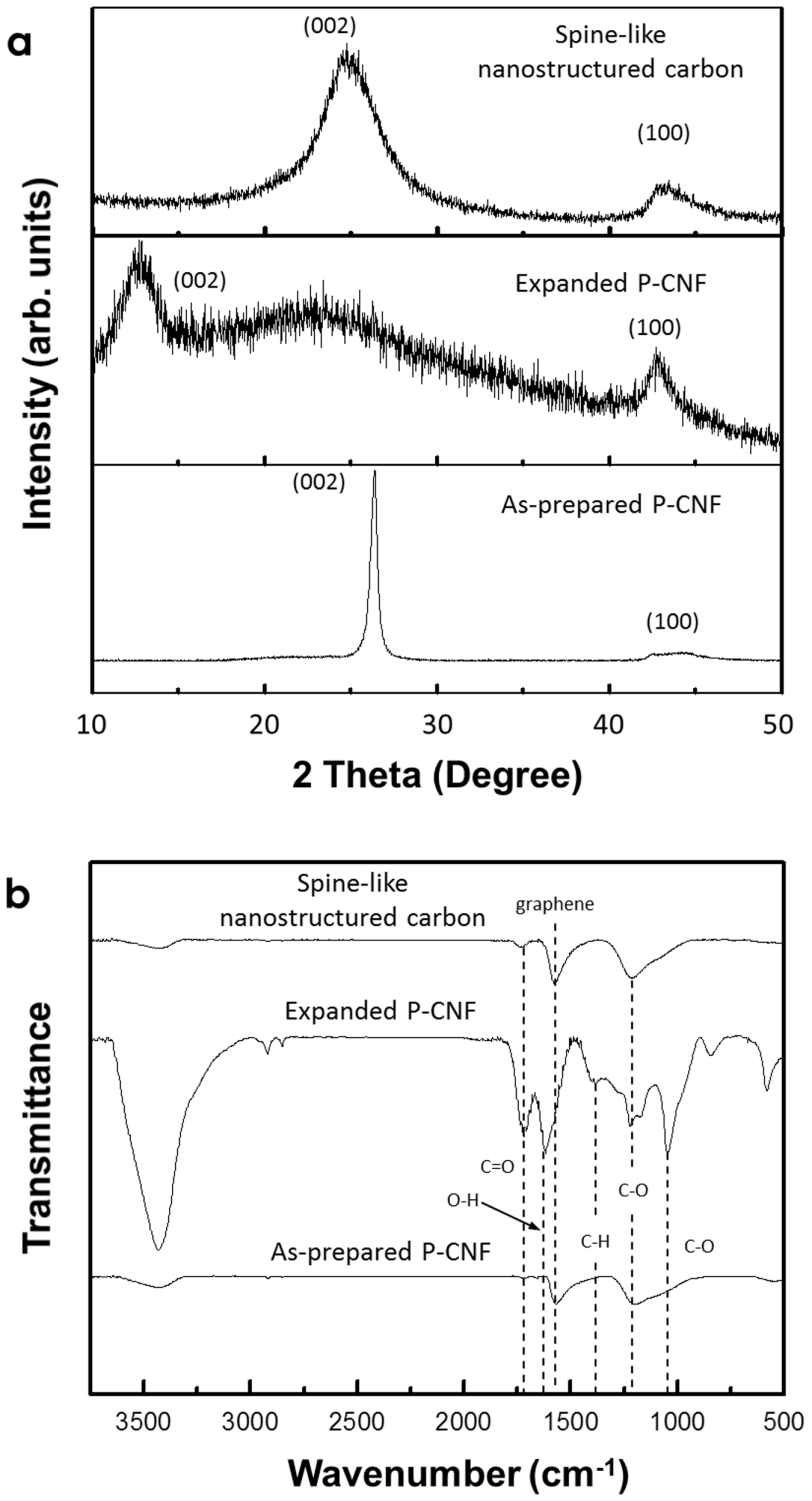
(a) XRD patterns and (b) FT-IR spectra for as-prepared P-CNF, expanded P-CNF and spine-like nanostructured carbon.

Structural changes in CNFs during synthesis were examined by X-ray diffraction (XRD). In the XRD pattern of as-prepared P-CNF (Figure 4a), the main diffraction peak observed at 2*θ* = 26° for the (002) plane corresponds to a highly oriented graphitic structure with an interlayer spacing of 0.34 nm^9^,^28^,^29^. The new peak at 2*θ* = 12°, which appears after expanding as-prepared P-CNF by optimized oxidation treatment, indicates an increase in the interlayer spacing (0.72 nm) due to the introduction of oxygen-containing functional groups^28^,^29^. However, the peak for expanded P-CNF is broader and shifted to a higher angle relative to that for GO prepared by Hummers' method (typically GO has a sharp peak at less than 10°)^28^,^29^. Most importantly, a broad hump remains around 2*θ* = 26°. These results suggest that the graphene interlayer spacing in expanded P-CNF does not increase uniformly; such non-uniform expansion could generate a periodically exfoliated graphitic structure rather than thinly chopped graphitic blocks during further exfoliation processing. For spine-like nanostructured carbon, a diffraction peak that appears at around 25° indicates a decrease in the interlayer spacing to ~0.36 nm due to the removal of oxygen-containing functional groups. However, the peak for spine-like nanostructured carbon is broader than that for as-prepared P-CNF, possibly because of the partially exfoliated graphitic structure (see TEM images above).

Fourier transform infrared (FT-IR) spectra demonstrated clear evidence for the introduction of oxygen-containing functional groups and their decomposition during the oxidation and reduction processes. Figure 4b shows the FT-IR spectra of as-prepared P-CNF, expanded P-CNF and spine-like nanostructured carbon. The as-prepared P-CNF exhibits characteristic peaks at 3440, 1570 and 1200 cm^−1^, which could be due to the H_2_O absorbed by the KBr pellets, and the skeletal vibrations of graphene and epoxy C–O, respectively^29^,^33^,^36^. For expanded P-CNF, new peaks corresponding to the oxygen-containing functional groups appeared at 1720, 1380 and 1040 cm^−1^, which were ascribed to the C = O stretching mode of the COOH group, carboxy C–O and alkoxy C–O, respectively^29^,^33^,^36^. The peak at 1620 cm^−1^ could be due to a combination of O–H bending vibration and epoxide groups^29^,^33^. Together with the XRD results, the FT-IR analyses suggest that the introduction of oxygen-containing functional groups during oxidation treatment increase the graphene interlayer spacing. For spine-like nanostructured carbon, signals from oxygen-containing functional groups such as C = O (1720 cm^−1^), carboxy C–O (1380 cm^−1^) and alkoxy C–O (1040 cm^−1^) decreased dramatically, and the peak at 1568 cm^−1^ from the skeletal vibration of graphene reappears.

For a more quantitative analysis of the oxidation/reduction level of CNFs during each processing step, the carbon/oxygen atomic ratio (C/O ratio) was evaluated by elemental analysis (see Supplementary Table S1). As-prepared P-CNF contains 97.47 wt.% C and 2.24 wt.% O. After expanding as-prepared P-CNF by oxidation treatment, the C/O ratio decreases to 3.05, which indicates the introduction of oxygen-containing functional groups. However, the C/O ratio for expanded P-CNF is still higher than previously reported values for GO prepared by Hummers' method^9^,^29^,^36^. (Effectively exfoliated RGO can typically be fabricated from highly oxidized GO with a C/O ratio below 2^29^,^36^.) Furthermore, it was reported that the graphitic blocks in CNF were effectively exfoliated and formed graphene nanodiscs when the C/O ratio of the oxidized CNF was below 2.29^28^. (also see Supplementary Figure S2 for thinly chopped graphitic blocks prepared from expanded CNF with a C/O ratio of 2.11) These results suggest that spine-like nanostructured carbon, which has periodically exfoliated graphitic blocks, is generated from expanded P-CNF by adjusting the degree of oxidation. Spine-like nanostructured carbon shows a C/O ratio of 13.23, which indicates reduction of expanded P-CNF and recovery of the π-conjugated electronic structure^37^. XPS results also support the change in CNF oxidation states with the introduction/decomposition of oxygen-containing function groups (see Supplementary Figure S5 and Table S2 for the quantitative determination of relative atomic percentages of sp^2^, sp^3^ carbons, and oxygen-functional groups).

**Figure 5:**
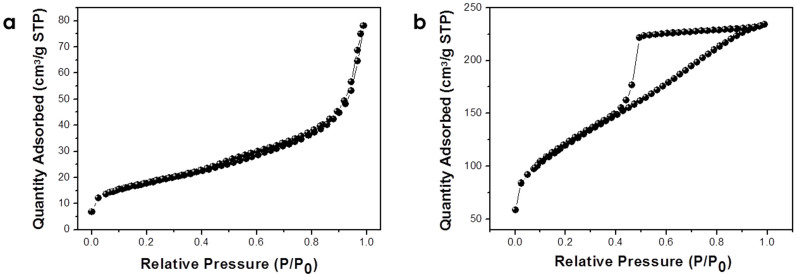
N_2_ adsorption–desorption isotherms of (a) as-prepared P-CNF (specific surface area (SSA): 64 m^2^/g) and (b) spine-like nanostructured carbon (SSA: 428 m^2^/g).

The electrochemical properties of carbon materials are highly dependent on their structural properties (surface area and pore characteristic) and electrical conductivity. Therefore, the specific surface areas and the pore size distributions of the as-prepared P-CNF and spine-like nanostructure carbon were measured by using the BET and BJH methods. The BET surface area of nanostructured carbon is 428 m^2^ g^−1^, which is significantly higher than that of as-prepared P-CNF (64 m^2^ g^−1^). In addition, spine-like nanostructured carbon exhibits a typical type-IV isotherm with H1 and H2 hysteresis loops, in correspondence with the characteristic isotherm for RGO (Figure 5)^9^,^37^. This result indicates the presence of exposed graphene layers between the graphitic blocks, layers that could highly increase the specific surface area of spine-like nanostructured carbon. The geometrically calculated surface areas are consistent with the possibility that the exposed graphene layers can increase the specific surface area in the resulting nanostructured carbon (see Supplementary Figure S6). More importantly, the spine-like nanostructured carbon maintained its initial specific surface area and structural properties (one dimensional shape and hierarchical structure) well even after solution processing and compression of the as-prepared powder (see Supplementary Figure S7), which could enable realization of large electrochemically active surface area under the electrode. The spine-like nanostructured carbon exhibits a narrow pore size distribution with an average pore diameter of 3.9 nm (see Supplementary Figure S8).

The electrical conductivities were measured by the two-point probe method with a compressed powder pellet (see Supplementary Figure S9)^37^. The electrical conductivity of as-prepared P-CNF was measured to be 1,283 S m^−1^, whereas it decreased to 388 S m^−1^ for spine-like nanostructured carbon, presumably due to the partially exfoliated graphitic structure. However, the electrical conductivity of the spine-like nanostructured carbon is still higher than that of other graphene-based carbon materials such as the microwave-hydrothermally synthesized RGO reported previously by our group (277 S m^−1^) and the herringbone-type CNF (139 S m^−1^, see Supplementary Figure S3)^37^. In contrast, the perfectly separated graphitic blocks prepared by the harsh exfoliation treatment (Figure 3d) show the lowest electrical conductivity (117 S m^−1^). This result demonstrates that the graphene nanoplatelets that interconnect the graphitic blocks support high electrical conductivity in spine-like nanostructured carbon.

**Figure 6:**
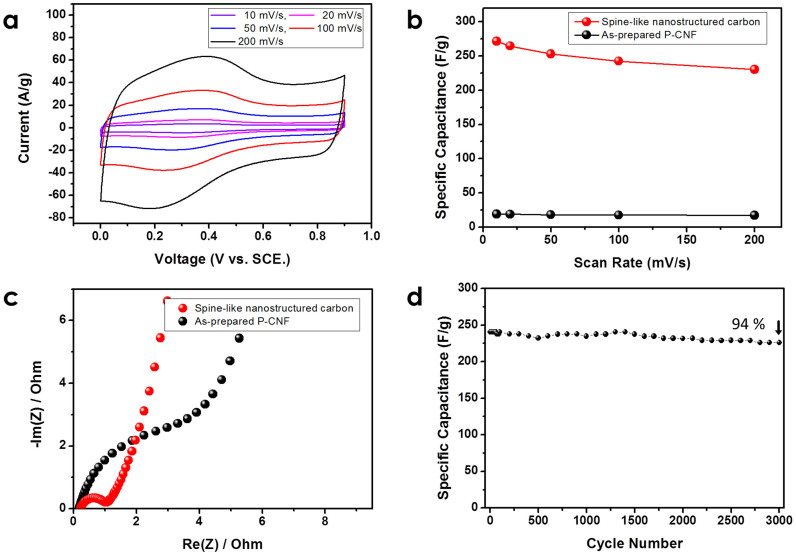
(a) Cyclic voltammograms at various scan rates for spine-like nanostructured carbon and (b) rate capabilities of as-prepared P-CNF and spine-like nanostructured carbon. (c) Nyquist plots for pristine P-CNF and spine-like nanostructured carbon. (d) Cycle stability of the spine-like nanostructured carbon measured at a scan rate of 100 mV/s.

Figure 6 shows the electrochemical properties of spine-like nanostructured carbon and as-prepared P-CNF. The electrode was composed of 90 wt.% active material (the P-CNF and spine-like nanostructured carbon) and 10 wt.% polyvinylidene fluoride (PVDF) binder. Conductive additives were not added to the electrode because of the high electrical conductivity of the nanostructured carbon itself. Figure 6a shows the cyclic voltammograms (CVs) of the spine-like nanostructured carbon electrode measured by using a three-electrode cell in the potential window between 0.0 and 0.9 V (*vs*. saturated calomel electrode (SCE)) in a 1 M H_2_SO_4_ electrolyte solution at a scan rate of 10 to 200 mV s^−1^. The quasi-rectangular shape of the CV at a scan rate of 10 mV s^−1^ indicates typical capacitive behavior due to the simultaneous electrical double-layer capacitance and pseudocapacitance^18^. When the scan rate increases to 200 mV s^−1^, the CV maintains the quasi-rectangular shape, indicating that the spine-like nanostructured carbon electrode shows good high-rate capability. Figure 6b shows the specific capacitance of the as-prepared P-CNF and spine-like nanostructured carbon electrodes calculated from the CVs for different scan rates. The specific capacitance of the spine-like nanostructured carbon electrode at a scan rate of 10 mV s^−1^ was calculated to be 272 F g^−1^, which is significantly higher than that of the as-prepared P-CNF electrode (19 F g^−1^ at 10 mV s^−1^). In the symmetric two-electrode cell using spine-like nanostructured carbon electrodes, the specific capacitance was measured to be 238.8 F/g based on a single-electrode by galvanostatic charge-discharge test at a current density of 2.5 A/g. This value is similar to that measured by CV for a three-electrode cell. (See Supplementary Figure S10 for the galvanostatic charge-discharge curves and the detailed calculation method) It is noteworthy that the specific capacitance of spine-like nanostructured carbon is one of the outstanding values reported for graphene-based carbon electrodes, even without further surface modifications such as those involving activation or chemical doping (see Supplementary Table S3)^18^,^19^,^20^,^21^,^22^,^23^,^24^,^25^,^26^,^38^,^39^,^40^,^41^,^42^,^43^,^44^. When the scan rate increased to 200 mV s^−1^, the specific capacitance of the spine-like nanostructured carbon electrode decreased to 230 F g^−1^, (above 85% of the initial value at 10 mV s^−1^), highlighting the excellent rate capability of spine-like nanostructure carbon.

Figure 6c shows the Nyquist plots for the as-prepared P-CNF and spine-like nanostructured carbon electrodes, as investigated by electrochemical impedance spectroscopy (EIS). Both Nyquist plots, which reveal a line along an imaginary axis at low frequencies, indicate typical capacitive behavior^18^. However, the Nyquist plots differ in shape at high frequencies. Unlike as-prepared P-CNF, spine-like nanostructured carbon clearly shows a semicircle at high frequencies, which is associated with the porous structure due to the partial exfoliation and the faradaic reaction (pseudocapacitance) of the residual oxygen functional groups^45^,^46^. The EIS result also suggests that pores are generated in spine-like nanostructured carbon owing to the partially exfoliated graphitic structure (see discussion of BET analysis in Figure 5).

The cycle stability of a supercapacitor is another important requirement for its practical application. Figure 6d shows the specific capacitance as a function of cycle number at a scan rate of 100 mV s^−1^. After 3000 cycles, the specific capacitance decreases by only 6% of initial specific capacitance, demonstrating that the spine-like nanostructured carbon electrode good cycle stability and a high degree of reversibility. (also see Supplementary Figure S11 for cycling performance in a symmetric two-electrode cell).

## Discussion

We have successfully synthesized a spine-like nanostructured carbon by combining an expanding process and a co-solvent exfoliation method. The co-solvent system induces mild exfoliation conditions that prevent disconnection of the graphene nanoplatelets between the graphitic blocks.

The spine-like nanostructured carbon shows two unique structural features. First, graphitic blocks of similar size (~100 nm) are periodically exfoliated along the major axis. Second, the exfoliated graphitic blocks are interconnected by thin graphene nanoplatelets in the manner of ligaments. Owing to these structural characteristics, the spine-like nanostructured carbon has a significantly higher specific surface area of 428 m^2^ g^−1^ than that of as-prepared P-CNF (64 m^2^ g^−1^), and mostly maintains its initial surface area after undergoing processes similar to real electrode fabrication. Additionally, the nanostructure maintains high electrical conductivity (388 S m^−1^) owing to the graphene nanoplatelet interconnections between graphitic blocks. The resultant spine-like nanostructured carbon exhibits significantly improved electrochemical performance in terms of specific capacitance (272 F g^−1^ at 10 mV s^−1^ in a three-electrode cell test and 238.8 F g^−1^ at 2.5 A/g in a two-electrode cell test) and rate capability (230 F g^−1^ at 200 mV s^−1^, above 85% of the initial value at 10 mV s^−1^).

The improved electrochemical performance of the spine-like nanostructured carbon can be explained as follows. In this nanostructured carbon, the partially exfoliated graphene nanoplatelets that are exposed to the electrode/electrolyte interface improve the double-layer capacitance of the electrode by increasing the electrochemically active surface area. Additionally, the partially residual, oxygen-containing functional groups on the exfoliated graphene layers undergo pseudocapacitive redox reactions and enhance the capacitance of the nanostructured carbon electrode. Furthermore, the graphitic blocks interconnected by graphene nanoplatelets in the carbon structure maintain the high electrical conductivity of spine-like nanostructured carbon, which likely supports the good rate capability of the electrode. Consequently, owing to the unique hierarchical structure of partially exfoliated sp2 graphitic blocks interconnected by graphene nanoplatelets, the spine-like nanostructured carbon electrode exhibits improved electrochemical performance in terms of capacitance and rate capability. Moreover, owing to its structural and electrochemical properties, this spine-like nanostructure carbon has significant potential as a high performance electrode material for other energy storage and conversion devices.

## Methods

The synthetic process to fabricate spine-like graphene-interconnected nanostructured carbon consists of three steps: preparation of P-CNF, an expanding process and an exfoliation and reduction process (Figure 1).

### Preparation of platelet-type carbon nanofiber by chemical vapor deposition

P-CNF was synthesized using a purified Fe metal catalyst with a carbon monoxide/hydrogen mixture gas^27^,^28^. The Fe metal catalyst was placed in a rotary furnace and reduced in H_2_/He (1:9 v/v) gas at 600°C for 1 h prior to the reaction with the carbon-containing mixture gas. Then the carbon monoxide/hydrogen mixture (4:1 v/v) gas was introduced at a flow rate of 2 L/min. The furnace was maintained at 600°C for 2 h while rotating at a speed of 5 cycles/min. After the reaction, the system was allowed to cool to room temperature. The obtained powder was then washed several times with HCl to remove the Fe metal catalyst.

### Preparation of expanded CNF by oxidation treatment

To expand the graphitic blocks in as-prepared P-CNF, we introduced a modified Hummers' method with concentrated sulfuric acid (H_2_SO_4_, 95%, Samchun Chemical), potassium permanganate (KMnO_4_, 99%, Aldrich) and hydrogen peroxide (H_2_O_2_, 35% in water, Junsei) in an ice bath^9^. A mixture containing 3 g of CNF and 250 ml of concentrated H_2_SO_4_ was stirred at room temperature. Subsequently, 3–18 g of KMnO_4_ was slowly added as an oxidizer to the CNF/H_2_SO_4_ mixture maintained at 0°C. By adjusting this mass ratio, 6 g of KMnO_4_ (KMnO_4_:CNF = 2:1) was found to be suitable for fabricating spine-like nanostructured carbon in the subsequent process (see Supplementary Figure S2). After vigorously stirring the P-CNF mixture for 2 h at 35°C, 500 mL of distilled water was added, and the resultant mixture was stirred for another hour. Then, 50 mL of H_2_O_2_ was added dropwise to the mixture until there was no gas evolution. The solid product was separated by centrifugation and washed several times with 0.1 M hydrochloric acid (HCl, 35–37%, Samchun Chemical) and distilled water to remove impurities. Finally, expanded P-CNF powder was obtained by freeze-drying overnight.

### Synthesis of spine-like graphene-interconnected nanostructured carbon via co-solvent exfoliation method and reduction processes

The third step involved the synthesis of spine-like nanostructured carbon from expanded P-CNF by a co-solvent exfoliation method and a subsequent reduction process. The expanded P-CNF powder was dispersed in a co-solvent that contained water (soluble solvent) and ethanol (insoluble solvent) at a ratio of 1:1. The concentration of the dispersion was 1 mg/ml, and subsequent exfoliation was conducted via ultrasonication by using a probe-type sonicator (VCX 750, Sonics & Materials Inc.) at a power of 400 W for 20 min at ~0°C. Furthermore, to promote homogeneous ultrasonication treatment and prevent locally intense forces, the dispersion was continuously stirred by a magnetic stirrer during exfoliation. For purposes of comparison, the expanded P-CNF powder was also exfoliated in water by ultrasonication treatment (harsh exfoliation condition). The sonication parameters (400 W, 20 min) in this condition were identical. To reduce CNF, hydrazine monohydrate (N_2_H_4_ 99%, Aldrich) was added to the dispersion and allowed to react at 80°C for 24 h. The solid product was filtered, washed with distilled water and freeze-dried overnight.

### Characterization of materials

The structural, chemical and electrical properties of as-prepared P-CNF, expanded P-CNF and spine-like nanostructured carbon were characterized by the following analytical procedures. The morphology and microstructure of CNFs were observed by high-resolution TEM (HR-TEM; JEM-3010, JEOL) and SEM (S-4300E, Hitachi). Structural changes in CNFs during synthesis were examined by XRD. Raman spectroscopy (LabRam HR, Horiba Jobin Yvon) was carried out to investigate the structural properties of CNFs, including disorder and defect structures. FT-IR spectra were obtained at room temperature between 4000 and 500 cm^−1^ at a spectral resolution of 4 cm^−1^ on a Perkin Elmer 1710 spectrophotometer by using KBr pellets. X-ray photoelectron spectroscopy (XPS) measurements were performed to investigate the oxidation and reduction states of CNFs by using an ESCA 2000 spectrometer (VG Microtech). Elemental analysis (Thermo EA1112, Thermo Electron Corp.) was also carried out to determine the chemical composition during each processing step. The surface area and pore characteristics of CNFs and spine-like nanostructured carbon were determined by analyzing the nitrogen gas adsorption isotherm with the Brunauer–Emmett–Teller (BET) method (ASAP ZOZO, Micromeritics Co.). The pore size distributions were derived from the desorption branches of the isotherms by using the Barrett–Joyner–Halenda (BJH) model. The electrical conductivities were measured for a powder pellet compressed in the form of a disc by using the two-point probe method in a poly(methyl methacrylate) (PMMA) cell.

The electrochemical properties at room temperature were investigated using three-electrode and symmetric two-electrode cells. The working electrode was prepared by slurry casting a mixture of 90 wt.% active material (as-prepared P-CNF, spine-like nanostructured carbon) and 10 wt.% PVDF dissolved in NMP as a binder onto Ti foil. Conductive additives such as carbon black were not added to the electrode owing to the high electrical conductivity of the CNF itself. The mass of electrode material coated onto the Ti foil was about 1 mg/cm^2^. In the three-electrode cell, we used a platinum plate as the counter electrode and an SCE as the reference electrode, and we conducted cyclic voltammetry (CV) in an aqueous electrolyte solution of 1 M H_2_SO_4_ by using a potentiostat–galvanostat (VMP3, Princeton Applied Research) in a potential window of 0.0–0.9 V vs. SCE. The specific capacitance of the electrode was calculated from the voltammetric charge of the CV curves. In addition, EIS measurements were carried out using an impedance analyzer (VMP3, Princeton Applied Research) at a DC bias voltage of 0.4 V vs. SCE in an AC frequency range of 200 kHz to 10 mHz with an AC amplitude of 5 mV. In the symmetric two-electrode cell, the specific capacitance was measured in 1 M H_2_SO_4_ electrolyte with a potential window of 0.0–0.9 V through a galvanostatic charge-discharge test.

## Author Contributions

S. Park and K. Kim designed the experiments. S. Yun and H. Jeong prepared the carbon nanofibers as the starting material. H. Kim and S. Yoon analyzed the electrochemical impedance spectroscopy of the electrodes. H. Park and J. Han participated in analyzing the electrochemical properties of the samples. J. Han, K. C. Roh and K. Kim reviewed and commented on the manuscript. S. Park wrote the manuscript. All authors discussed the results and commented on the manuscript.

## Supplementary Material

Supplementary Information Supplementary Information
