# Supplementary material for: Spine-like Nanostructured Carbon Interconnected by Graphene for High-performance Supercapacitors
Source: Sci Rep. 2014 Aug 19;4:6118. doi: 10.1038/srep06118 (PMC4137264; doi:10.1038/srep06118)
Supplement: Supplementary Information [file srep06118-s1.doc]

Supplementary Information

Spine-like Nanostructured Carbon Interconnected by Graphene for High-performance Supercapacitors

Sang-Hoon Park^1^, Seung-Beom Yoon^1^, Hyun-Kyung Kim^1^, Joong Tark Han^2^, Hae-Woong Park^3^, Joah Han^3^, Seok-Min Yun^4^, Han Gi Jeong^4^, Kwang Chul Roh^3^ & Kwang-Bum Kim^1^

^1^Department of Materials Science and Engineering, Yonsei University, 134 Shinchon-dong, Seodaemoon-gu, Seoul 120-749, Republic of Korea, ^2^Nano Carbon Materials Research Group, Korea Electrotechnology Research Institute, Changwon 642-120, Republic of Korea, ^3^Energy Efficient Materials Team, Energy & Environmental Division, Korea Institute of Ceramic Engineering & Technology, 233-5 Gasan-dong, Guemcheon-gu, Seoul 153-801, Republic of Korea, ^4^VINA Tech, 587-40, Gwahaksaneop 2-ro, Ochang-eup, Cheongwon-gun, Chungcheongbuk-do, 63-883, Republic of Korea.

**Contents**

**Figure S1.** High-magnification TEM images of as-prepared P-CNF and spine-like nanostructured carbon.

**Figure S2.** XRD patterns for expanded P-CNFs prepared at different mass ratios and resulting nanostructure after exfoliation treatment

**Figure S3.** TEM images of as-prepared H-CNF.

**Figure S4.** Raman spectra of P-CNF, expanded P-CNF and spine-like nanostructured
carbon.

**Figure S5.** C 1s XPS spectra of the as-prepared P-CNF, expanded P-CNF, and spine-like
nanostructured carbon.

**Figure S6.** Schematic diagrams of as-prepared P-CNF and spine like nanostructured carbon.

**Figure S7.** Spine-like nanostructured carbon after solution processing, compression, and preparation of an electrode by using the slurry coating method.

**Figure S8.** BJH pore size distributions of the as-prepared P-CNF and spine-like nanostructured carbon.

**Figure S9.** Schematic diagram of cell used for measuring electrical conductivity of
samples.

**Figure S10.** Charge-discharge curves of the symmetric two-electrode cell using spine-like nanostructured carbon electrodes.

**Figure S11.** Cycling performance of the symmetric two-electrode cell.

**Table S1.** Elemental analysis of as-prepared P-CNF, expanded P-CNF, spine-like
nanostructured carbon and as-prepared H-CNF.

**Table S2.** Analysis of C1s peak position and the relative atomic percentages of sp^2^, sp^3^, and oxygen functional groups for the samples.

**Table S3.** Comparison of electrochemical performances of graphene-based carbon electrodes.

**Figures**

**
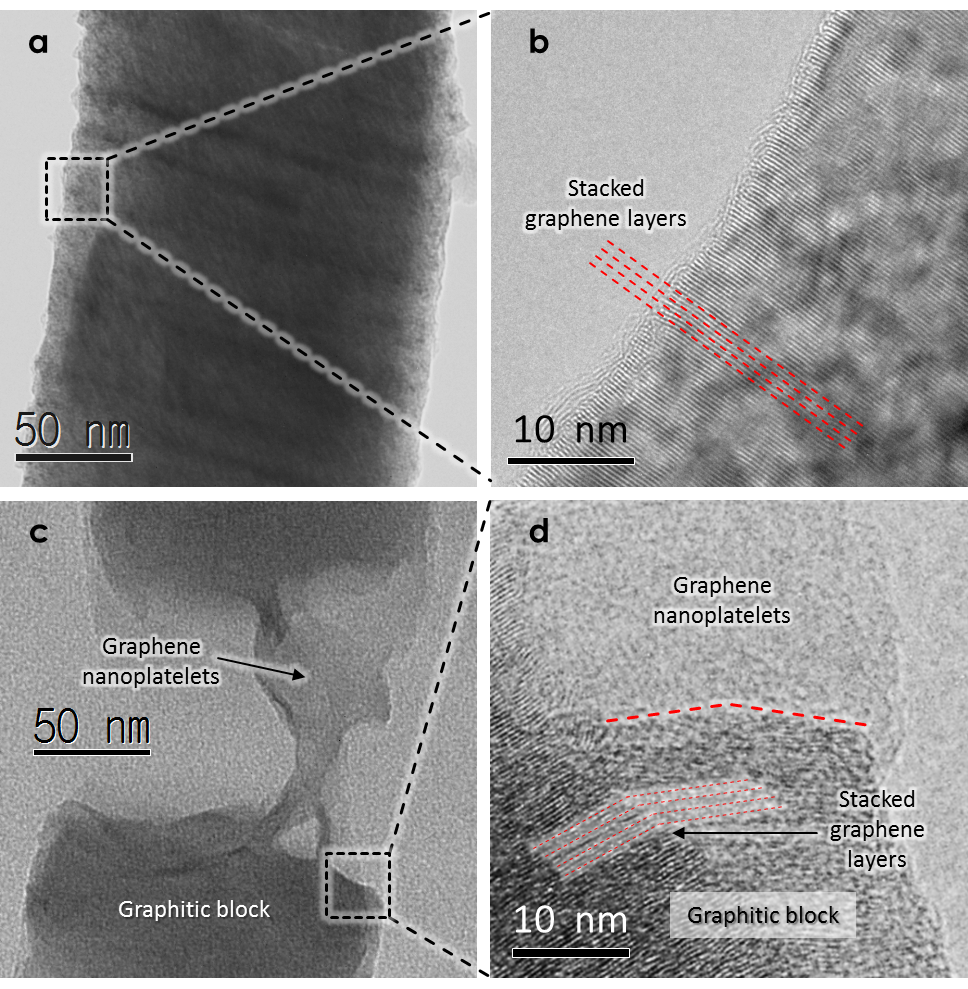
**

**Figure S1.** TEM images of (a) as-prepared P-CNF and (b) edge structure of as-prepared P-CNF at high magnification. TEM images of (c) spine-like nanostructured carbon and (d) graphitic block interconnected by graphene nanoplatelets at high magnification.

Figure S1 shows high-magnification TEM images of as-prepared P-CNF and spine-like nanostructured carbon. In as-prepared P-CNF (Figure S1a and S1b), the graphene layers are stacked perpendicular to the fiber axis and their edges are exposed in the radially outward direction of the fiber. Figure S1c shows that graphitic blocks were partially exfoliated but kept the graphene nanoplatelets interconnected to form spine-like nanostructured carbon. In the high-magnification image of Figure S1d, graphene nanoplatelets are interconnected with a graphitic block that consists of graphene layers stacked perpendicular to the major axis.


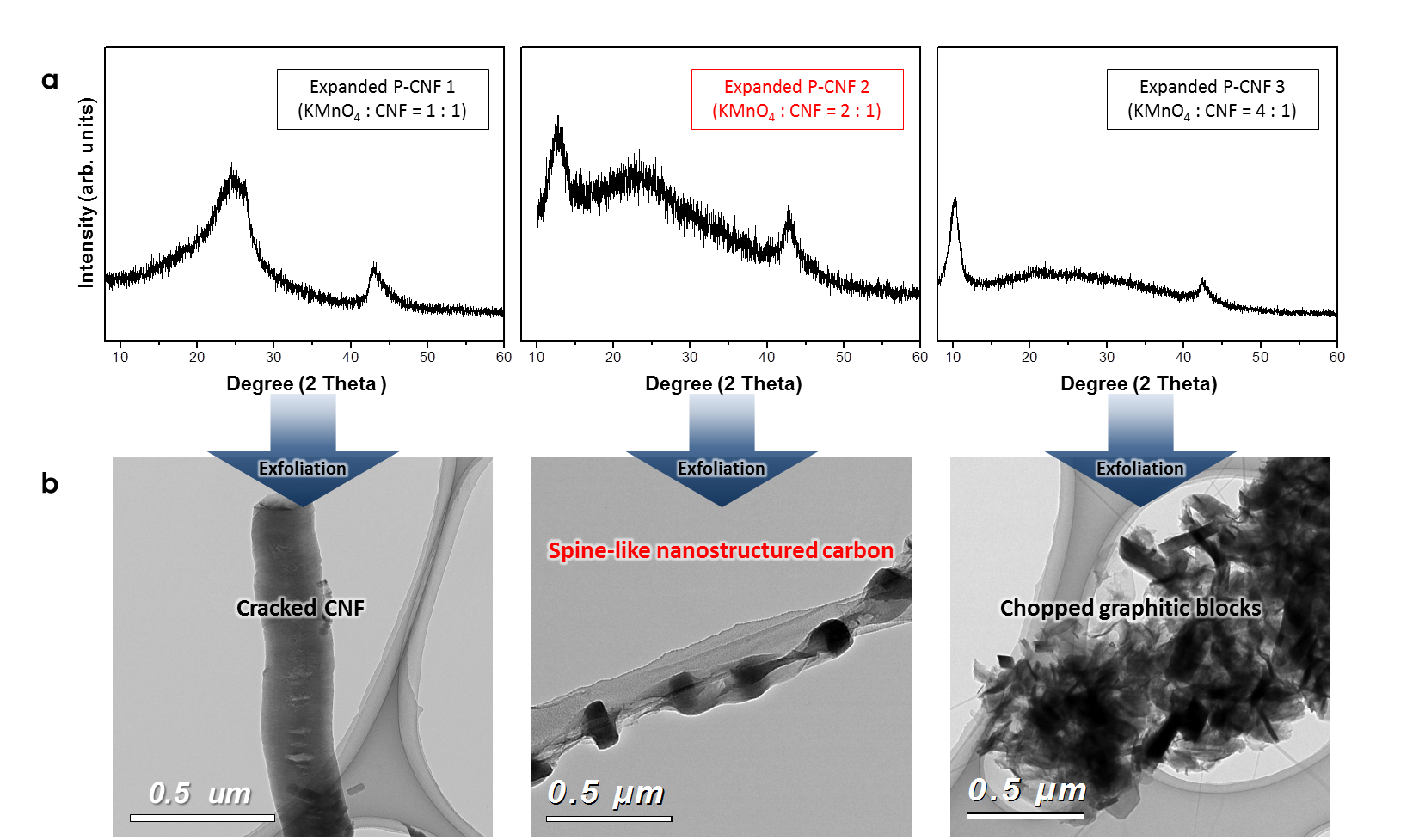


**Figure S2.** (a) XRD patterns for expanded P-CNFs prepared at different mass ratios of the KMnO_4_ oxidant to CNF during oxidation treatment. (b) TEM images of resulting nanostructured carbons after exfoliation treatment.

The degree of oxidation of the expanded-CNF greatly affected the morphology of the nanostructured carbon after exfoliation treatment. Therefore, we sought to find the optimized oxidation condition for expanded P-CNF by varying the mass ratio of the KMnO_4_ oxidant to CNF during oxidation treatment. Figure S2a shows the XRD patterns for expanded P-CNFs prepared by different mass ratios of KMnO_4_ to CNF (1:1, 2:1 and 4:1). The expanded P-CNF 1 (KMnO_4_:CNF = 1:1) exhibits a broad peak at 2*θ* = 24°, indicating that the interlayer spacing of the graphene layers was not substantially increased during oxidation treatment. In contrast, with a mass ratio of 2:1 (expanded P-CNF 2), a new broad peak appears at 12° with a broad hump around 26°; at a mass ratio of 4:1 (expanded P-CNF 3), the broad hump decreases dramatically and the main peak is observed at 2θ = 10°. The degree of sample oxidation could be quantitatively confirmed by elemental analysis. The C/O ratios obtained by elemental analysis for expanded P-CNF 1, 2 and 3 were 7.77, 3.05 and 2.11, respectively.

Figure S2b shows the TEM images of the resulting nanostructured carbons prepared from these expanded P-CNFs after exfoliation treatment. In the case of expanded P-CNF 1 (C/O ratio of 7.77), the graphitic structure in CNF is partially cracked but not exfoliated along the fiber axis after exfoliation treatment. For expanded P-CNF 2 with a proper degree of oxidation (C/O ratio of 3.05), spine-like nanostructured carbon could be formed during further co-solvent exfoliation treatment. (The detailed results are discussed in the manuscript. The expanded P-CNF 2 with a proper degree of oxidation was normally denoted as “expanded P-CNF” in this study.) However, for expanded P-CNF 3 with a high degree of oxidation (C/O ratio of 2.11), most of the graphitic blocks were thinly chopped even after the same exfoliation condition.

**
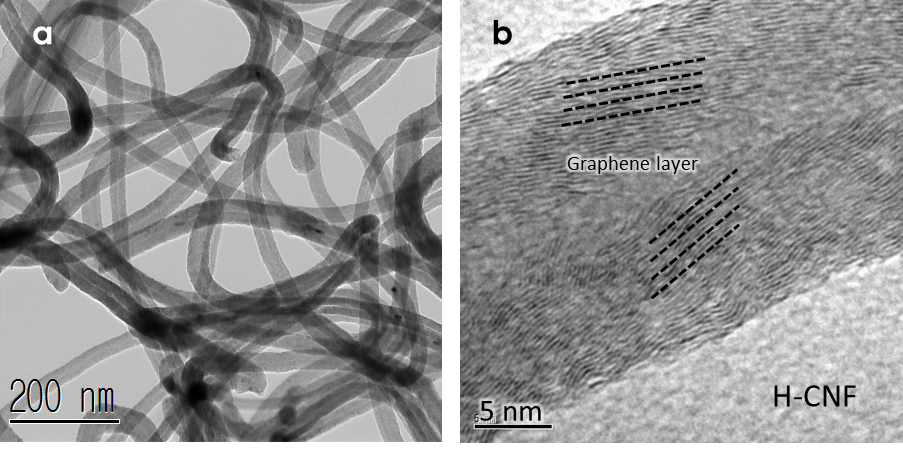
**

**Figure S3.** TEM images of as-prepared H-CNF at (a) low and (b) high magnifications.

For a comparison of electrical properties, herringbone-type CNF (H-CNF; average diameter of 40 nm) was also prepared and characterized. H-CNF was synthesized using a Ni/Fe metal catalyst with carbon monoxide/hydrogen gas at 700 °C.^[1–3]^ To remove the metal catalyst, H-CNF powder was washed several times with HCl. Figure S3a shows TEM images of as-prepared H-CNF. Compared to as-prepared P-CNF, H-CNF has a narrower fiber diameter (40 nm) and higher surface area (147 m^2^ g^-1^). In H-CNF, the graphene layers are stacked in a wedge-like formation perpendicular to the fiber axis. The electrical conductivity of as-prepared H-CNF is 139 S m^-1^, which is lower than that of both as-prepared P-CNF and spine-like nanostructured carbon synthesized in this study.


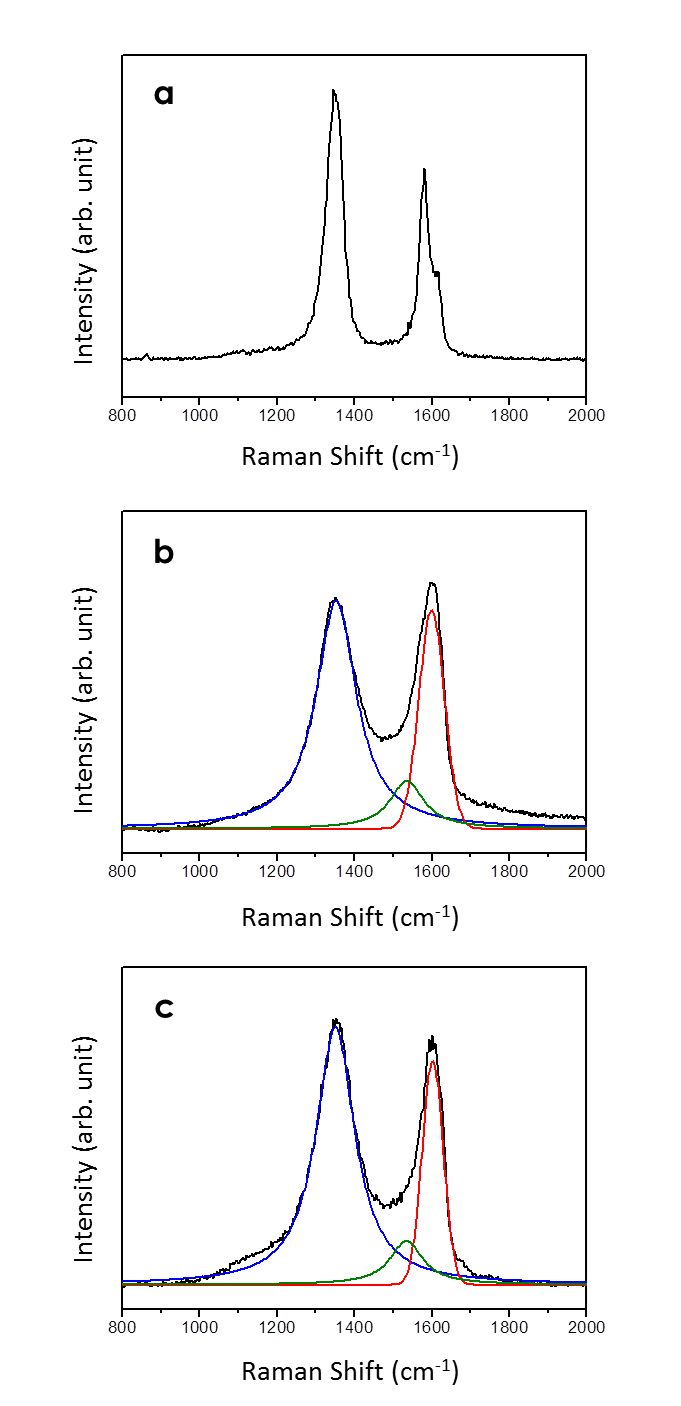


**Figure S4.** Raman spectra of P-CNF, expanded P-CNF and spine-like nanostructured carbon.

Figure S4 shows the Raman spectra of as-prepared P-CNF, expanded P-CNF and spine-like nanostructured carbon. All spectra show two distinct peaks at 1350 and 1580 cm^-1^, which correspond to the D and G bands, respectively.^[4–9]^ The D band originates from the disorder-induced mode associated with defects or lattice disorders (*e*.*g*., non-sp^2^ composition), whereas the G band corresponds to first-order scattering of the E2g mode from the sp^2^ carbon domains. The Raman spectrum of as-prepared P-CNF (Figure S4a) shows that the D band peak is higher than the G band peak. The D band is sensitive not only to graphitic defects within the graphene basal plane but also edge atoms on the graphene edge plane.^[9,11,12]^ The graphene planes of P-CNF are perpendicular to the fiber axis, and the outer surface of P-CNF is mainly occupied by edge atoms.^[4–9]^ This could explain why the D/G ratio of P-CNF is higher than that of other graphitic carbon materials. After expanding P-CNF by oxidation treatment (Figure S4b), a broad band around 1500 cm^-1^, characteristic of amorphous carbon, appears between the D and G bands.^[4–9]^ To quantitatively evaluate amorphous carbon together with disordered and ordered sp^2^ graphitic carbon, the Raman spectra were fitted using a mixed Gaussian–Lorentzian curve.^[4,5,7,8]^ The spectrum of spine-like nanostructured carbon (Figure S4c), which shows a D/G ratio (1.10) that is higher than that of expanded P-CNF (0.94), indicates that spine-like nanostructured carbon has more defect structures than does the expanded P-CNF. This result, which may reflect the degradation of graphitic crystallinity due to exfoliation, is similar to that reported for chemically reduced GO.^[5,10]^

**
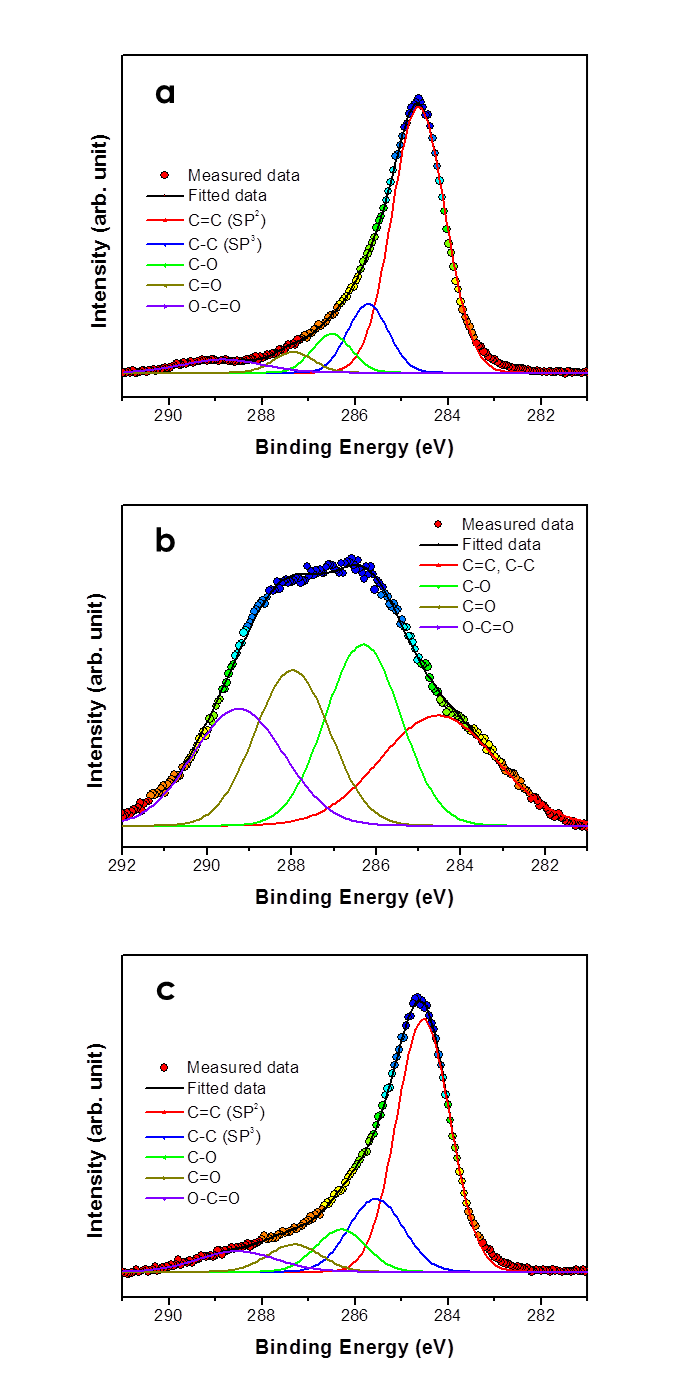
**

**Figure S5.** C 1s XPS spectra of (a) as-prepared P-CNF, (b) expanded P-CNF and (c) spine-like nanostructured carbon.

Figure S5 shows the C1s spectra of the as-prepared P-CNF, expanded P-CNF, and spine-like nanostructured carbon, which consist of five typical components arising from C=C (sp^2^, ~284.6 eV), C-C (sp^3^, ~285.5 eV), C-O (hydroxyl and epoxy, ~286.5 eV), C=O (carbonyl, ~288.3 eV), and O-C=O (carboxyl, 289.0 eV), as described previously.^[13-14]^ Table S2 lists the quantitative determination of the relative atomic percentages of the sp^2^, sp^3^ carbons, and oxygen-functional groups, which were evaluated by fitting of the XPS 1Cs peak. The as-prepared P-CNF possesses 83.1% of sp^2^/sp^3^ carbon components with 16.9% of hetero-carbon components arising from oxygen-functional groups such as C-O, C=O, and O-C=O. After expanding the P-CNF by oxidation treatment, the percentage from the hetero-carbon components significantly increased to 72.8%. For spine-like nanostructured carbon prepared by co-solvent exfoliation and reduction process, the percentage of sp^2^/sp^3^ carbon components increased to 76.9%, and the hetero-carbon components decreased to 23.1%. However, hetero-carbon components such as C-O, C=O, and O-C=O are slightly higher than those for as-prepared P-CNF, which might be due to some residual oxygen-containing functional groups that were introduced on the exfoliated graphene nanoplatelets.^[13, 15]^ The partially residual, oxygen-containing functional groups on the exfoliated graphene layers undergo pseudocapacitive redox reactions and enhance the capacitance of the spine-like nanostructured carbon electrode.^[16-17]^


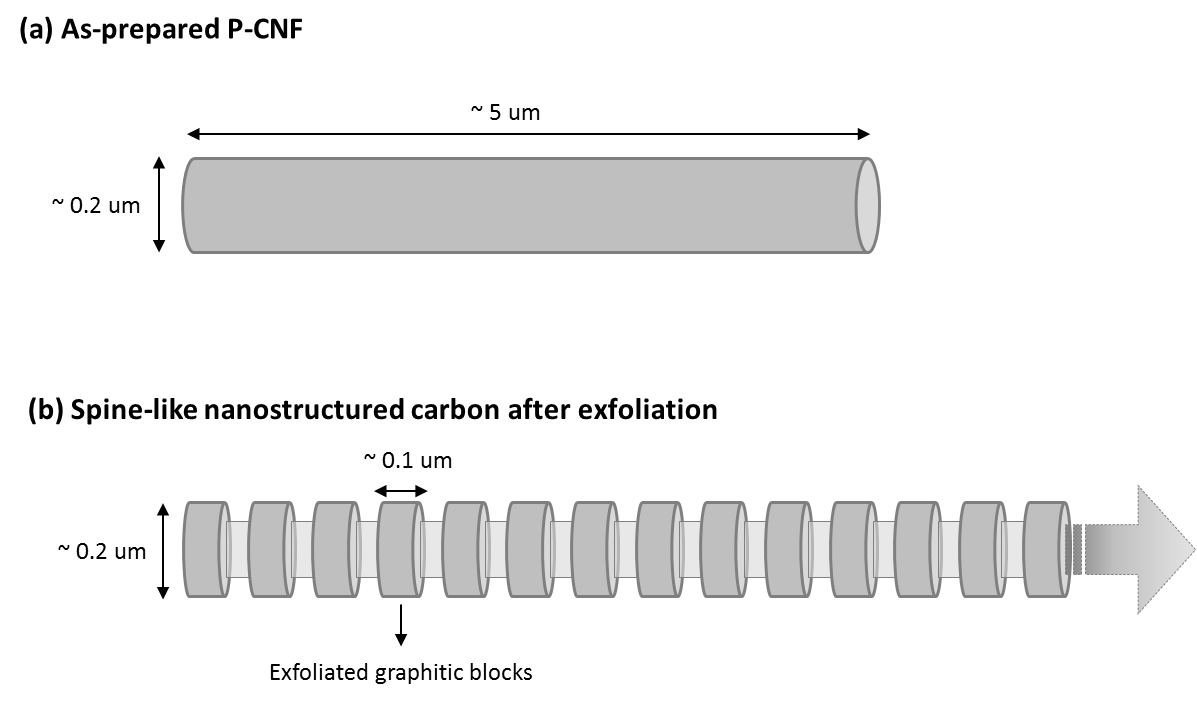


**Figure S6.** Schematic diagrams of an as-prepared P-CNF and spine-like nanostructured carbon.

The geometrically calculated surface areas obtained from simplified structures are consistent with the view that exposure of the graphene layers contributes significantly to the increased specific surface area in the resulting spine-like nanostructured carbon. To calculate the surface area, we assume that the P-CNFs have a cylindrical shape. The average length and diameter of the P-CNFs are considered to be 5 and 0.2 µm, respectively, from the TEM and SEM observations. We further assume that spine-like nanostructured carbon consists of several exfoliated graphitic blocks. The average size (length along the major axis) and diameter of the exfoliated graphitic blocks are considered to be 0.1 and 0.2 µm, respectively. The number of blocks is considered to be 50 by dividing the initial length of a P-CNF by the size of the graphitic blocks. Figure S6 shows schematic diagrams of a P-CNF and a spine-like nanostructured carbon as based on the above-mentioned assumptions.

The surface area of a P-CNF (SA _PCNF_) can be calculated as follows:

SA _PCNF_ = (π · r^2^) × 2 + (π · 2 r) × L = 3.204 µm^2^,

where r is the fiber radius and L is the fiber length. To facilitate the calculations, the weight of the P-CNFs is not considered.

The surface area of a spine-like nanostructured carbon (SA _spine_) is then calculated by using the total sum of the surface area of exfoliated graphitic blocks. In this case, the surface area of the exposed graphene nanoplatelets between graphitic blocks was not considered.

SA _spine_ = {(π · r'^2^) × 2 + (π · 2 r') × L'} × N = 6.283 µm^2^

Here, r' is the block radius, L' is the block size and N is the number of blocks.

From these calculations, it can be observed that the geometrically calculated surface area approximately doubles after exfoliation. However, the specific surface areas of the P-CNF and spine-like nanostructured carbon as measured by BET analysis were 64 and 428 m^2^ g^-1^, respectively. The actually measured increment in the specific surface area is much higher than the geometrically calculated value. This might be attributed to the presence of the exposed graphene platelets between the graphitic blocks in the spine-like nanostructured carbon. The partially exfoliated graphene nanoplatelets could have significantly increased the surface area of the resulting nanostructured carbon.

Although these calculations do not provide a quantitative value of the specific surface area of graphene nanoplatelets in the nanostructured carbon, they do show that the graphene nanoplatelets could significantly increase the surface area of the resulting nanostructured carbon.


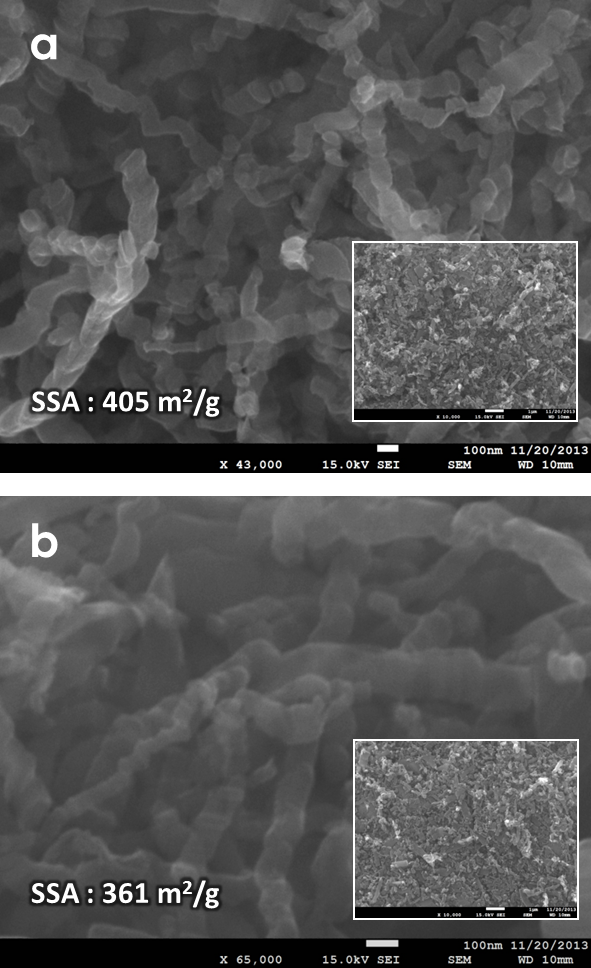


**Figure S7.** SEM images of the spine-like nanostructured carbon (a) after solution processing and (b) after compression.

The surface area under the powder state does not seem to directly determine the electrochemical performance of electrode for supercapacitor applications. More importantly, the surface area of the electrode materials should be maintained even after real electrode fabrication process to achieve the large electrochemically active surface area under the electrode state. Accordingly, in order to investigate the change in the surface area during the electrode fabrication process, we conducted additional experiments: (i) solution processing (solvent impregnation and drying) and (ii) compression of the powder, according to the previous report by Huang *et al*.^18^ (Because of the effect of the binder and the presence of the current collector, it is difficult to directly investigate the change in the surface area of the electrode materials during the electrode fabrication process by using the slurry coating method. Thus, we measured the specific surface area of the electrode materials in the powder form during these additional processes.)

Figure S7 shows the SEM images of the spin-like nanostructured carbon after solution processing (the powder was dispersed in ethanol and then dried at 80°C in conventional oven) and compression of the powder (the powder was compressed under constant pressure of 2000 psi for 10 min). The spine-like nanostructured carbon maintained its initial one-dimensional shape well after solution processing (Figure S7a) and compression (Figure S7b) of the as-prepared powder. More importantly, the specific surface area of the spine-like nanostructured carbon decreased slightly after these processes (initial specific surface area: 428 m^2^/g; after solution processing: 405 m^2^/g; and after compression: 361 m^2^/g). This might be a result of the structural properties of the spine-like nanostructured carbon (one-dimensional shape and the hierarchical structure with graphitic block/graphene nanoplatelets). While 2D graphene nanosheets were easily stacked into a planar paper-like structure under the additional processes, the spine-like nanostructured carbon was not tightly stacked by the formation of the entangled web-like structure from its one-dimensional shape. (See Ref. 18: Their regular r-GO prepared by thermal shock initially possessed the specific surface area of 407 m^2^/g. After solution processing or compression of the as-prepared r-GO powder, the specific surface area decreased to 226 and 65 m^2^/g, respectively.) Furthermore, the graphitic blocks between nanoplatelets act as a pillar to prevent the re-stacking of the partially exfoliated graphene nanoplatelets, which could better maintain its specific surface area during the additional processes. We believe that this is the main reason for the better performance in term of specific capacitance compared to that of the conventional high surface area graphene

**
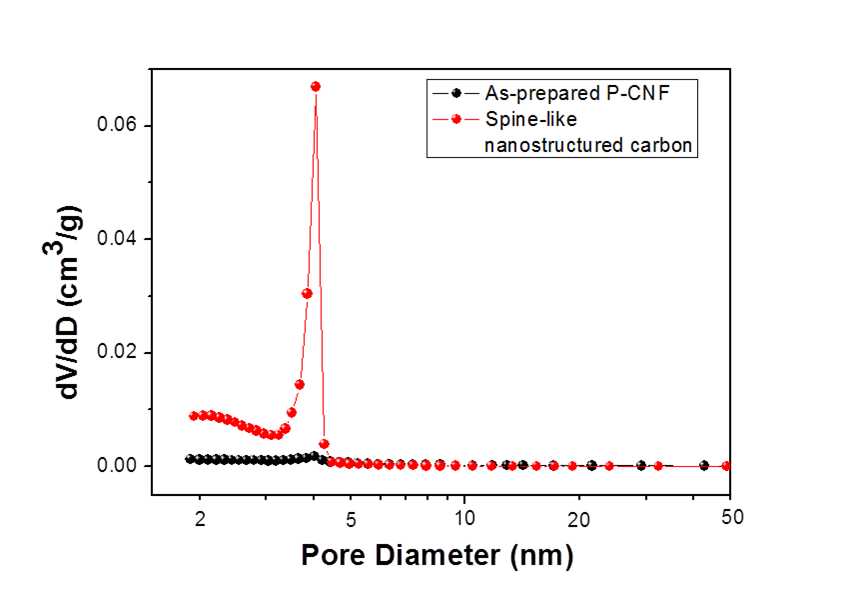
**

**Figure S8.** BJH Pore size distributions of as-prepared P-CNF and spine-like nanostructured carbon.

Figure S8 shows the Barrett–Joyner–Halenda (BJH) pore size distributions of the as-prepared P-CNF and spine-like nanostructured carbon. The quantity of the dV/dD pore volume of the spine-like nanostructured carbon is significantly higher than that of the as-prepared P-CNF. This might be due to the exfoliated graphene layers between the graphitic blocks in the resulting nanostructured carbon. Additionally, the spine-like nanostructured carbon exhibits a narrow pore size distribution with an average pore diameter of 3.9 nm, similar to the characteristic pore size distribution for graphene-based carbon materials (RGO, CNT, RGO/CNT hybrid, and exfoliated graphitic carbon).^[19-23]^ The nano-sized pores for spine-like nanostructured carbon could enable fast and efficient ion access, leading to high-power performance in supercapacitor cells.


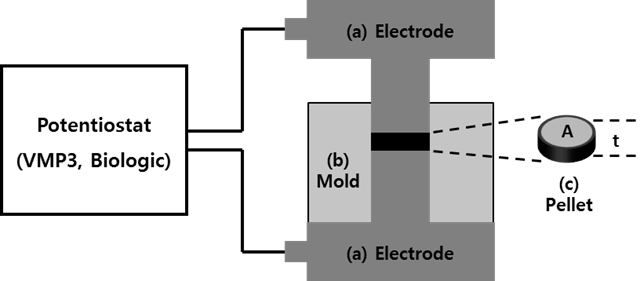


**Figure S9.** Schematic diagram of cell used for measuring the electrical conductivity of samples by the 2-point probe method. (a) Stainless steel top electrode and fixed bottom electrode; (b) poly(methyl methacrylate) (PMMA) mold; and (c) carbon pellet with area A and thickness t (electrode area = 1.33 cm^2^).

The electrical conductivities were measured with a powder pellet compressed in the form of a disc by using the two-point probe method in a poly(methyl methacrylate) (PMMA) cell (Figure S9).^[10]^ CNF powder was compressed under a constant pressure of 8000 psi, and the thickness of the resulting pellet was measured using a digital micrometer.


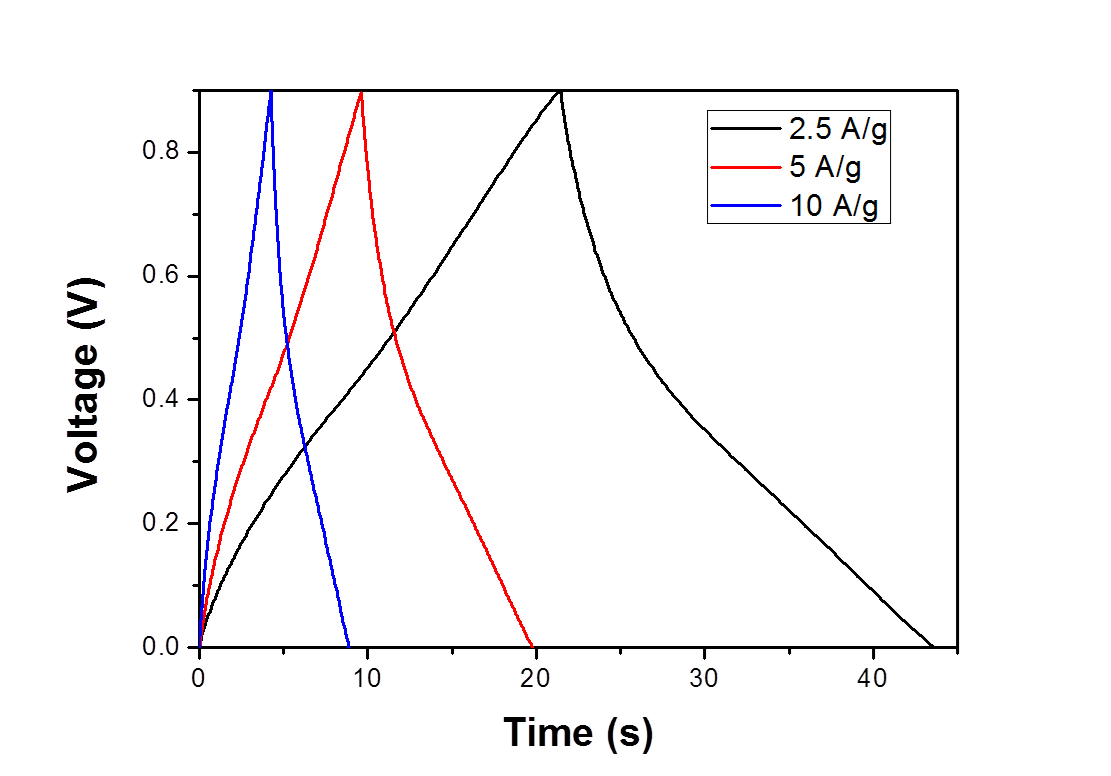


**Figure S10.** Charge-discharge curves of the symmetric two-electrode cell using the spine-like nanostructured carbon electrodes at current densities of 2.5–10 A/g.

Figure S10 shows the charge-discharge curves of the symmetric two-electrode cell using the spine-like nanostructured carbon electrodes at current densities of 2.5–10 A/g. The specific capacitance of a single-electrode (C_single-electrode_) in the symmetric two-electrode cell was calculated from the galvanostatic charge-discharge curve according to the equation:

C_single-electrode_ = 4 × I/(m dV/dt)

where C_single-electrode_, I, m, and dV/dt indicate the specific capacitance for a single-electrode, the constant applied current, the total mass of both electrodes, and the slope obtained from discharge curve, respectively.^24, 25^ The specific capacitance for a single electrode (C_single-electrode_) for spine-like nanostructured carbon was measured to be 238.4 F/g at 2.5 A/g, which was almost similar with the value measured by CV in the three-electrode cell (230 F/g a scan rate of 200 mV/s). When the current density increased to 10 A/g, the specific capacitance decreased to 197.6 F/g.

**
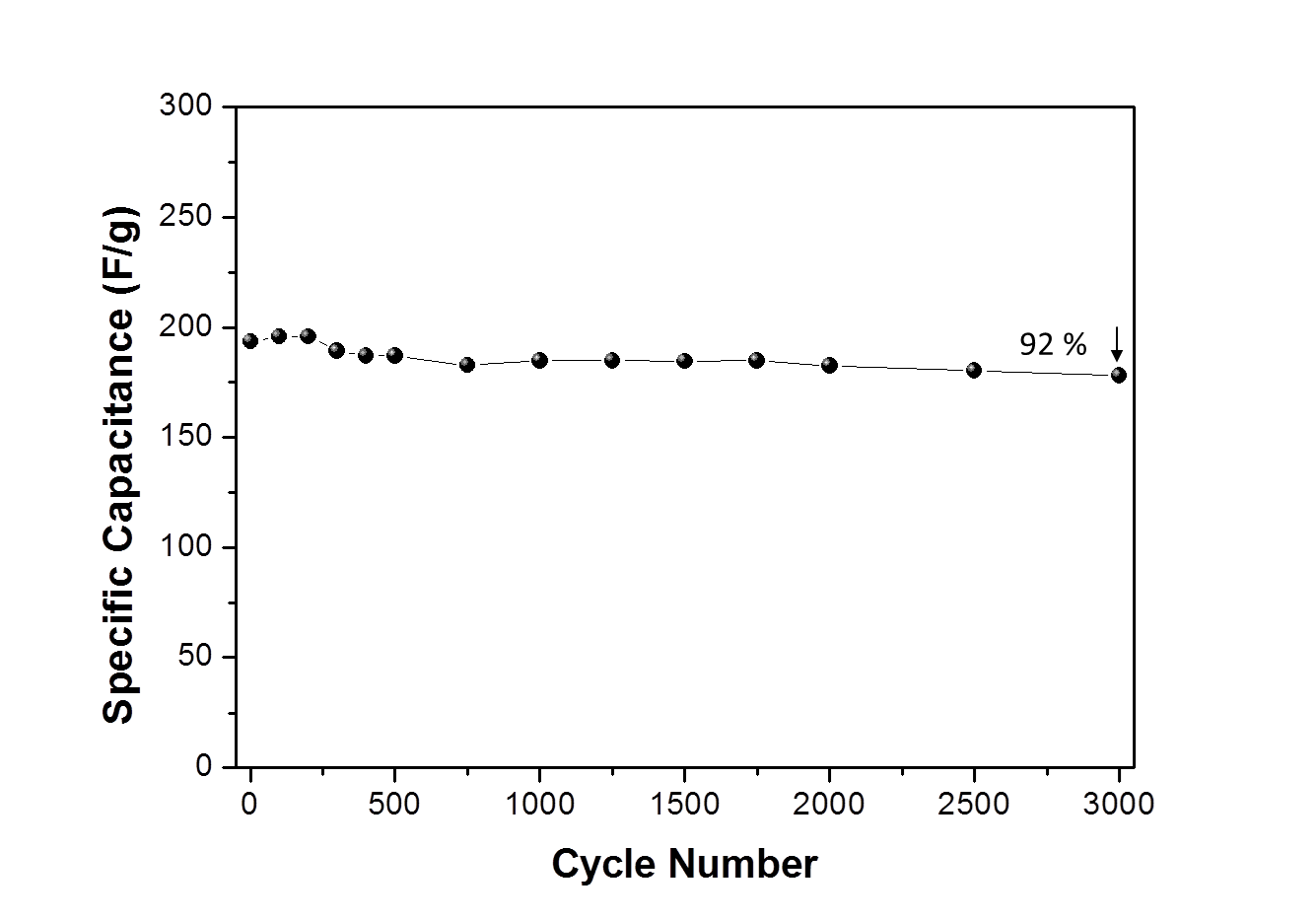
**

**Figure S11.** Cycling performance of the spine-like nanostructured carbon measured by galvanostatic charge-discharge method in a symmetric two-electrode cell. The cycling performance of symmetric two-electrode cell was evaluated between 0.0–0.9 V at a current density of 10 A/g in H_2_SO_4_ aqueous electrolyte for 3000 cycles.

Table S3 lists the electrochemical performances of our spine-like nanostructured carbon and the graphene-based electrode materials reported in the literature. All reference data shown in Table S3 is based on **the specific capacitance of a single-electrode** (C_single-electrode_) measured by a two-electrode cell test using an aqueous electrolyte, such as KOH or H_2_SO_4_. Upon comparing our spine-like nanostructured carbon electrode with other graphene-based electrodes, we would like to emphasize that the specific capacitance of the spine-like nanostructured carbon (238.8 F/g at 2.5 A/g) exceeds many of the excellent values reported for graphene-based carbon electrodes (usually 120~200 F/g in other studies), and furthermore, this value was achieved without additional surface modifications, such as processes involving activation or chemical doping. Additionally, when comparing the other electrochemical performances, such as rate capability and cyclability, our spine-like nanostructured carbon exhibits good rate capability (197.6 F/g at 10 A/g) with competitive cyclability (92% retention after 3000 cycles) among the graphene-based carbon materials.

**Table S1.** Elemental analysis of as-prepared P-CNF, expanded P-CNF, spine-like nanostructured carbon, and as-prepared H-CNF. The carbon/oxygen atomic ratio (C/O ratio) was calculated from the obtained data for more quantitative analyses.

| Sample | C/wt.% | O/wt.% | H/wt.% | S/wt.% | N/wt.% | C/O ratio |
| --- | --- | --- | --- | --- | --- | --- |
| As-prepared  P-CNF | 97.47 | 2.24 | 0.29 | - | - | 58.08 |
| Expanded P-CNF | 67.08 | 29.29 | 2.26 | 1.32 | 0.06 | 3.05 |
| Spine-like nanostructured carbon | 88.51 | 8.92 | 0.92 | 1.25 | 0.18 | 13.23 |

**Table S2.** Analysis of C1s peak position and the relative atomic percentages of sp^2^, sp^3^, and oxygen functional groups for the as-prepared P-CNF, expanded P-CNF, and spine-like nanostructured carbon.

| Sample | C=C | C−C | C−O | C=O | O−C=O |
| --- | --- | --- | --- | --- | --- |
| As-prepared P-CNF | 284.6 (69.3 %) | 285.5 (13.8 %) | 286.4 (7.5 %) | 287.3 (4.2 %) | 288.9 (5.2 %) |
| Expanded P-CNF | 284.6 (26.1 %) | | 286.3 (28.0 %) | 287.8 (23.8 %) | 289.2 (22.%) |
| Spine-like nanostructured carbon | 284.6 (59.4 %) | 285.5 (17.5 %) | 286.3 (9.4 %) | 287.3 (6.5 %) | 288.6 (7.2 %) |

**Table S3.** Comparison of electrochemical performances for graphene-based carbon electrodes.

*All reference values are based on C_single-electrode_ measured by a two-electrode cell test.

| Electrode material | Test method | Electrochemical performances  (C_single-electrode_)* | Ref. |
| --- | --- | --- | --- |
| Graphene (RGO) | 2-electrode cell  Galvanostatic method  (5.5 M KOH) | 135 F/g at 0.01 A/g  128 F/g at 0.02 A/g | 26  (R. Ruoff *et al.*) |
| Graphene/CNT | 2-electrode cell  Galvanostatic method (1 M H_2_SO_4_) | 120 F/g at 0.1 A/g  ~60 F/g at 8 A/g  98% retention after 10,000 cycles | 27  (D. Mitlin *et al.*) |
| Graphene/ activated carbon | 2-electrode cell Galvanostatic method (KOH) | 122 F/g at 0.1 A/g  ~90 F/g at 4 A/g  90% retention after 3,000 cycles | 28  (Y. Ma *et al.*) |
| Graphene/carbon black pillared structure | 2-electrode cell  Cyclic voltammetry (6 M KOH) | 138 F/g at 10 mV/s  82 F/g at 500 mV/s  94% retention after 2,000 cycles | 29  (J. Lian *et al.*) |
| Vertically oriented graphene | 2-electrode cell  Galvanostatic method (6 M KOH) | 140 F/g at 1 A/g  104% retention after 2,900 cycles | 30  (J. Chen *et al.*) |
| Flat/crumpled graphene hybrid | 2-electrode cell  Galvanostatic method (5 M KOH) | 181 F/g at 0.1 A/g  135 F/g at 2.5 A/g  ~99% retention after 5,000 cycles | 31  (J. Huang *et al.*) |
| 3D Hexaporous carbon/graphene | 2-electrode cell  Galvanostatic method (1 M H_2_SO_4_) | 154 F/g at 0.5 A/g  123 F/g at 10 A/g  ~78% retention after 1,000 cycles | 32  (S. Ogale *et al.*) |
| 3-D graphene/CNT | 2-electrode cell  Galvanostatic method (30 wt.% KOH) | 187 F/g at 0.1 A/g  167 F/g at 1 A/g  ~95% retention after 1,000 cycles | 33  (Y. Chen *et al.*) |
| 3D flexible graphene hydrogel film | 2-electrode cell  Galvanostatic method (1 M H_2_SO_4_) | 190 F/g at 1 A/g  152 F/g at 20 A/g  82% retention after 10,000 cycles | 34  (X. Dual *et al.*) |
| Graphene/CNT | 2-electrode cell  Cyclic voltammetry (6 M KOH) | 233 F/g at 50 mV/s  ~150 F/g at 500 mV/s  99% retention after 85,000 cycles | 35  (C. Ozkan *et al.*) |
| Assembled graphene | 2-electrode cell  Galvanostatic method (5 M KOH) | 222 F/g at 1 A/g  165 F/g at 100 A/g  92% retention after 2,000 cycles | 36  (G. Shi *et al.*) |
| 3D strutted graphene | 2-electrode cell  Galvanostatic method  (1 M H_2_SO_4_) | 250 F/g at 0.1 A/g  ~230 F/g at 1A/g | 37  (Y. Bando *et al*.) |
| **Spine-like nanostructured carbon** | 2-electrode cell  Galvanostatic method  (1 M H_2_SO_4_) | **238.8 F/g at 2.5 A/g**  **197.6 F/g at 10 A/g**  92% retention after 3,000 cycles | **Our work** |
| N-doped graphene | 2-electrode cell  Galvanostatic method (6 M KOH) | 282 F/g at 1 A/g  ~210 F/g at 10 A/g  95% retention after 10,000 cycles | 38  (J. Choi *et al.*) |

**References**

[1] Park, C., Engel, E. S., Crowe, A., Gilbert, T. R., Rodriguez, N. M. Use of Carbon Nanofibers in the Removal of Organic Solvents from Water. Langmuir 16, 8050 (2000).

[2] Yoon, S. H., Park, C. W., Yang, H., Korai, Y., Mochida, I., Baker, R. T. K., Rodriguez, N. M. Novel carbon nanofibers of high graphitization as anodic materials for lithium ion secondary batteries. Carbon 42, 21 (2004).

[3] Long, D., Hong, J. Y., Li, W., Miyawaki, J., Ling, L. C., Mochida, I., Yoon, S. H., Jang, J. Fabrication of Uniform Graphene Discs via Transversal Cutting of Carbon Nanofibers. ACS Nano 5, 6254 (2011).

[4] Li, P., Zhao, T. J., Zhou, J. H., Sui, Z. J., Dai, Y. C., Yuan, W. K. Characterization of carbon nanofiber composites synthesized by shaping process. Carbon 43, 2701 (2005).

[5] Jeon, I. Y., Shin, Y. R., Sohn, G. J., Choi, H. J., Bae, S. Y., Mahmood, J., Jung, S. M., Seo, J. M., Kim, M. J., Chang, D. W., Dai, L. M., Baek, J. B. Edge-carboxylated graphene nanosheets via ball milling. Proc. Natl. Acad. Sci. USA 109, 5588 (2012).

[6] Cancado, L. G., Pimenta, M. A., Neves, B. R. A., Dantas, M. S. S., Jorio, A. Influence of the Atomic Structure on the Raman Spectra of Graphite Edges. Phys. Rev. Lett. 93, 247401 (2004).

[7] Zheng, J. S., Zhang, X. S., Li, P., Zhou, X. G., Yuan, W. K. Microstructure effect of carbon nanofiber on electrocatalytic oxygen reduction reaction. Catal. Today 131, 270 (2008).

[8] Vollebregt, S., Ishihara, R., Tichelaar, F. D., Hou, Y., Beenakker, C. I. M. Influence of the growth temperature on the first and second-order Raman band ratios and widths of carbon nanotubes and fibers. Carbon 50, 3542 (2012).

[9] Pimenta, M. A., Dresselhaus, G., Dresselhaus, M. S., Cancado, L. G., Jorio, A., Saito, R. Studying disorder in graphite-based systems by Raman spectroscopy. Phys. Chem. Chem. Phys. 9, 1276 (2007).

[10] Park, S. H., Bak, S. M., Kim, K. H., Jegal, J. P., Lee, S. I., Lee, J., Kim, K. B. Solid-state microwave irradiation synthesis of high quality graphene nanosheets under hydrogen containing atmosphere. J. Mater. Chem. 21, 680 (2011).

[11] Nethravathi, C., Rajamathi, M. Chemically modified graphene sheets produced by the solvothermal reduction of colloidal dispersions of graphite oxide. Carbon 46, 1994 (2008).

[12] Liang, Y. Y., Wu, D. Q., Feng, X. L., Mullen, K. Dispersion of graphene sheets in organic solvent supported by ionic interactions. Adv. Mater. 21, 1679 (2009).

[13] Jeong, S. Y., Kim, S. H., Han, H. T., Jeong, H. J., Yang, S., Lee, G. W. High-Performance Transparent Conductive Films Using Rheologically Derived Reduced Graphene Oxide. ACS Nano 5, 870 (2011).

[14] Huang, Y. L., Tien, H. W., Ma, C. C., Yang, S. Y., Wu, S. Y., Liu, H. Y., Mai, Y. W. Effect of extended polymer chains on properties of transparent graphene nanosheets conductive film. J. Mater. Chem. 21, 18236 (2011).

[15] Bargri, A., Mattevi, C., Acik, M., Chabal, Y. J., Chhowalla, M., Shenoy, V. B. Structural evolution during the reduction of chemically derived graphene oxide. Nature Chem. 2, 581 (2010).

[16] Fan, X., Lu, Y., Xu, H. Kong, X., Wang, J. Reversible redox reaction on the oxygen-containing functional groups of an electrochemically modified graphite electrode for the pseudo-capacitance. J. Mater. Chem. 21, 18753 (2011).

[17] Lin, Z., Liu, Y., Yao, Y., Hildreth, O. J., Li, Z., Moon, K., Wong, C. P. Superior Capacitance of Functionalized Graphene. J. Phys. Chem. C115, 7120 (2011)

[18] Luo, J., Jang, H. D., Sun, T., Xiao, L. He, Z., Katsoulidis, A. P., Kanatzidis, M. G., Gibson, J. M., Huang, J. Compression and Aggregation-Resistant Particles of Crumpled Soft Sheets. ACS Nano 5, 8943 (2011)

[19] Liu, C., Yu, Z., Neff, D., Zhamu, A., Zang, B. J. Graphene-Based Supercapacitor with an Ultrahigh Energy Density. Nano Lett. 10, 4863 (2010).

[20] Xiang, H. F., Li, Z. D., Jiang, J. Z., Chen, J. J., Lian, P. C., Wu, J. S., Yu, Y., Wang, H. H. Graphene sheets as anode materials for Li-ion batteries: preparation, structure, electrochemical properties and mechanism for lithium storage. RSC Adv. 2, 6792 (2012).

[21 Wang, S., Haldane, D., Liang, R., Smithyman, J., Zhang, C. Wang, B. Nanoscale infiltration behaviour and through-thickness permeability of carbon nanotube buckypapers. Nanotechnology 24, 015704 (2013).

[22] Zhang, D., Yan, T., Shi, L., Peng, Z., Wen, X., Zhang, J. Enhanced capacitive deionization performance of graphene/carbon nanotube composites. J. Mater. Chem. 22, 14696 (2012)

[23] Long, D., Li, W., Miyawak, J., Qiao, W., Ling, L. Mochida, I. Yoon, S. H. Meso-channel Development in Graphitic Carbon Nanofibers with Various Structures. Chem. Mater. 23, 4141 (2011)

[24] Qu, D., Shi, H. Studies of activated carbons used in double-layer capacitors. J. Power Sources 74, 99 (1998).

[25] Stoller, M. D., Ruoff, R. S. Best practice methods for determining an electrode material's performance for ultracapacitors. Energy Environ. Sci. 3, 1294 (2010).

[26] Stoller, M. D., Park, S. J., Zhu, Y. W., An, J. H., Ruoff, R. S. Graphene-Based Ultracapacitors. Nano Lett. 8, 3498 (2008).

[27] Xu, Z. W., Li, Z., Holt, C. M. B., Tan, X. H., Wang, H. L., Amirkhiz, B. S., Stephenson, T., Mitlin, D. Electrochemical Supercapacitor Electrodes from Sponge-like Graphene Nanoarchitectures with Ultrahigh Power Density. J. Phys. Chem. Lett. 3, 2928 (2012).

[28] Chen, Y., Zhang, X., Zhang, H. T., Sun, X. Z., Zhang, D. C., Ma, Y. W. High-performance supercapacitors based on a graphene-activated carbon composite prepared by chemical activation. Rsc Adv. 2, 7747 (2012).

[29] Wang, G. K., Sun, X., Lu, F. Y., Sun, H. T., Yu, M. P., Jiang, W. L., Liu, C. S., Lian, J. Flexible Pillared Graphene-Paper Electrodes for High-Performance Electrochemical Supercapacitors. Small 8, 452 (2012).

[30] Bo, Z., Wen, Z. H., Kim, H., Lu, G. H., Yu, K. H., Chen, J. H. One-step fabrication and capacitive behavior of electrochemical double layer capacitor electrodes using vertically-oriented graphene directly grown on metal. Carbon 50, 4379 (2012).

[31] Luo, J. Y., Jang, H. D., Huang, J. X. Effect of Sheet Morphology on the Scalability of Graphene-Based Ultracapacitors. Acs Nano 7, 1464 (2013).

[32] Yadav, P., Banerjee, A., Unni, S., Jog, J., Kurungot, S., Ogale, S. A. 3D Hexaporous Carbon Assembled from Single-Layer Graphene as High Performance Supercapacitor. Chemsuschem 5, 2159 (2012).

[33] Wang, Y., Wu, Y. P., Huang, Y., Zhang, F., Yang, X., Ma, Y. F., Chen, Y. S. Preventing Graphene Sheets from Restacking for High-Capacitance Performance. J. Phys. Chem. C 115, 23192 (2011).

[34] Xu, Y. X., Lin, Z. Y., Huang, X. Q., Liu, Y., Huang, Y., Duan, X. F. Flexible Solid-State Supercapacitors Based on Three-Dimensional Graphene Hydrogel Films. Acs Nano 7, 4042 (2013).

[35] Wang, W., Guo, S. R., Penchev, M., Ruiz, I., Bozhilov, K. N., Yan, D., Ozkan, M., Ozkan, C. S. Three dimensional few layer graphene and carbon nanotube foam architectures for high fidelity supercapacitors. Nano Energy 2, 294 (2013).

[36] Zhang, L., Shi, G. Q. Preparation of Highly Conductive Graphene Hydrogels for Fabricating Supercapacitors with High Rate Capability. J. Phys. Chem. C 115, 17206 (2011).

[37] Wang, X. B., Zhang, Y. J., Zhi, C. Y., Wang, X., Tang, D. M., Xu, Y. B., Weng, Q. H., Jiang, X. F., Mitome, M., Golberg, D., Bando, Y. Three-dimensional strutted graphene grown by substrate-free sugar blowing for high-power-density supercapacitors. Nat. Commun. 4, (2013).

[38] Jeong, H. M., Lee, J. W., Shin, W. H., Choi, Y. J., Shin, H. J., Kang, J. K., Choi, J. W. Nitrogen-doped graphene for high-performance ultracapacitors and the importance of nitrogen-doped sites at basal planes. Nano Lett. 11, 2472 (2011).
